# Investigating Virus–Host Interactions in Cultured Primary Honey Bee Cells

**DOI:** 10.3390/insects12070653

**Published:** 2021-07-17

**Authors:** Alexander J. McMenamin, Fenali Parekh, Verena Lawrence, Michelle L. Flenniken

**Affiliations:** 1 Department of Plant Sciences and Plant Pathology, Montana State University, Bozeman, MT 59717, USA; alexmcmenamin2@gmail.com (A.J.M.); fenali10@gmail.com (F.P.); verena.cml@gmail.com (V.L.); 2 Department of Microbiology and Cell Biology, Montana State University, Bozeman, MT 59717, USA; 3 Pollinator Health Center, Montana State University, Bozeman, MT 59717, USA

**Keywords:** honey bee, *Apis mellifera*, primary cell culture, insect antiviral defense, honey bee viruses, RNA viruses, insect viruses

## Abstract

**Simple Summary:**

Honey bees are eusocial insects that live in colonies comprised of ~30,000 individuals. They are the primary pollinators of plants that produce fruits, nuts, and vegetables. High annual losses of honey bee colonies have made it challenging for beekeepers to provide enough colonies to meet the demand for pollination services. Multiple stressors, including viruses, contribute to colony deaths. To better understand viral pathogenesis and bee antiviral defense mechanisms, we obtained primary cells from honey bee larvae and pupae, maintained them in culture, and infected them with a panel of viruses. We determined that larval hemocytes, which are primary immune cells, support replication of a honey bee virus (sacbrood virus) and a model virus (Flock House virus). Similarly, we determined that mixed cell populations derived from honey bee pupae support replication of sacbrood virus, Flock House virus, and another honey bee virus (deformed wing virus). Evaluation of the host cellular level response to infections with each of these viruses revealed unique expression profiles of three immune genes. In summary, this study demonstrates the utility of honey bee primary cell cultures to investigate the impacts of virus infection on honey bees at the cellular level, which in turn affects individual bee and colony health.

**Abstract:**

Honey bee (*Apis mellifera*) health is impacted by viral infections at the colony, individual bee, and cellular levels. To investigate honey bee antiviral defense mechanisms at the cellular level we further developed the use of cultured primary cells, derived from either larvae or pupae, and demonstrated that these cells could be infected with a panel of viruses, including common honey bee infecting viruses (i.e., sacbrood virus (SBV) and deformed wing virus (DWV)) and an insect model virus, Flock House virus (FHV). Virus abundances were quantified over the course of infection. The production of infectious virions in cultured honey bee pupal cells was demonstrated by determining that naïve cells became infected after the transfer of deformed wing virus or Flock House virus from infected cell cultures. Initial characterization of the honey bee antiviral immune responses at the cellular level indicated that there were virus-specific responses, which included increased expression of *bee antiviral protein-1* (GenBank: MF116383) in SBV-infected pupal cells and increased expression of *argonaute-2* and *dicer-like* in FHV-infected hemocytes and pupal cells. Additional studies are required to further elucidate virus-specific honey bee antiviral defense mechanisms. The continued use of cultured primary honey bee cells for studies that involve multiple viruses will address this knowledge gap.

## 1. Introduction

All organisms are infected by viruses, which are obligate intracellular pathogens that rely on host cell machinery to replicate. In turn, hosts evolve mechanisms to prevent and/or limit virus infections. To investigate host–virus interactions at the cellular and molecular levels, scientists rely extensively on cell cultures, including immortalized cell lines and primary cells [1,2,3]. Cultured cells are amenable to techniques that augment or silence gene expression (e.g., transfection of plasmids and nucleic acids) and are thus invaluable to studies aimed at elucidating biological mechanisms. Furthermore, cell cultures are also utilized to produce pure virus stocks, which are important for experimentation and vaccine production [4,5,6,7]. In spite of their utility and importance, robust cell culture systems are not available for many non-model organisms, including honey bees (*Apis mellifera*). While approximately 1000 cell lines have been derived from insect tissues, including 19 hymenopteran cell lines (the insect order that includes bees, ants, and wasps), only one immortalized honey bee cell line, AmE-711, has been established [8,9,10].

The development of additional tools to understand honey bee host–virus interactions at the cellular level is critical for determining the impact of virus infections at the individual bee and colony levels and may ultimately result in the development of strategies that reduce the number of virus-associated honey bee colony deaths. Honey bee colony losses averaged 38% annually from 2010 to 2018, in part due to viral infections [11,12,13,14,15,16,17,18,19]. Honey bees are cosmopolitan agricultural pollinators that are impacted by numerous stressors, including agrochemicals, lack of quality forage and habitat, colony and habitat management, the *Varroa destructor* mite, and pathogens including bacteria, fungi, trypanosomatids, and viruses [20,21,22,23,24,25,26,27,28,29,30,31]. The largest group of honey-bee-infecting pathogens are small RNA viruses (reviewed in [23,32]). The list of honey bee-infecting and honey-bee-associated viruses is ever growing, in part due to virus discovery efforts aided by the increased use of high throughput sequencing of either total RNA or virally enriched RNA sample sequencing [33,34,35,36,37,38,39,40,41] (reviewed in [23,24]). The most ubiquitous and well-studied honey-bee-associated viruses include acute bee paralysis virus [42], black queen cell virus [43], chronic bee paralysis virus [44,45], deformed wing virus [46,47], Israeli acute paralysis virus [48], Kashmir bee virus [49], sacbrood virus [50], and the Lake Sinai virus group [40,51]. Viral infections can result in various symptoms, from paralysis to deformity or shortened lifespan, but individual bees can also harbor high viral loads (~100 billion copies per bee) and show no apparent symptoms [40,52,53,54].

The *Iflaviridae* family comprises several arthropod-associated viruses, including two honey-bee-infecting viruses (i.e., sacbrood virus and deformed wing virus) [55,56,57,58]. Sacbrood virus (SBV) infects both larvae and adults and is likely spread throughout a colony by adult nurse bees that ingest infected larvae, develop SBV infections, including in their hypopharyngeal glands, and transmit the virus to new larvae by feeding them contaminated brood food [59,60]. While SBV may shorten the lifespan of adult bees, it is most pathogenic to young larvae, which become discolored and yellow as the infection progresses. As the infected larva develops, its cuticle becomes leathery, which prevents molting, and fluid builds up, creating a saclike appearance [58]. Concurrent with larvae production, SBV prevalence typically peaks in honey bee colonies in the spring and summer months [40,61,62,63,64]. Deformed wing virus (DWV) infects both developing and adult bees. Infection of larvae or pupae can cause wing deformities, likely due to infection of nascent wing buds [65,66,67,68]. DWV infection of adults is associated with short, bloated abdomens, shortened life span, and precocious foraging, while reducing total foraging time [69,70,71,72]. Infection of the brain is sometimes associated with increased aggression, reduced associative learning, and downregulation of genes associated with neuronal signaling, suggesting a dysregulation of normal brain function [73,74,75]. Deformed wing virus can be acquired vertically through contamination of eggs via infected ovarian tissue or DWV-positive semen or horizontally via mating, contaminated food (i.e., brood food or pollen), trophallaxis, pupal cannibalism, or the bite of a *Varroa destructor* mite [76,77,78,79,80,81] (reviewed in [82]). Varroa mites, which primarily feed on the honey bee fat body, serve as passive and/or replication-competent viral vectors [66,83,84,85,86,87]. DWV strains include DWV-A, DWV-B/VDV-1, which share approximately 84% nucleotide identity, and DWV-C, as well as recombinant viruses [88,89,90,91,92,93]. DWV is one of the most well-studied honey bee viruses, and high DWV levels, coupled with mite infestation, are associated with honey bee colony losses [94,95,96,97,98]. Recent development of DWV infectious clones will further our understanding of this virus and its interactions with bee and mite hosts [65,78,99]. In the absence of infectious clones, scientists have utilized crude virus preparations obtained from infected honey bees, including at the larval and pupal stages [10,68,86,100,101,102,103,104].

Model viruses, including Sindbis virus and Flock House virus, have also been utilized to investigate honey bee antiviral defense mechanisms [4,105,106,107,108,109]. These viruses are relatively easily to propagate and purify and are important tools for investigating antiviral response across a range of different species, including honey bees, fruit flies, and mosquitos [4,105,106,107,108,109,110,111,112]. Flock House virus (FHV) is a small, nonenveloped, icosahedral insect virus in the *Nodaviridae* family. FHV is genetically and structurally well-characterized and has been extensively utilized in studies aimed at understanding basic viral processes, including genome replication and virion assembly [113,114,115,116,117,118,119,120]. FHV is also a valuable model virus for investigating insect antiviral immunity. Although FHV was originally isolated from grass grubs (*Costelytra Zealandica*), it infects a range of insects, including mosquitoes, fruit flies, tsetse flies, and honey bees [105,106,110,111,121,122]. To counteract the host immune defense systems, several invertebrate viruses have evolved viral suppressors of RNAi (VSR), and RNA-based strategies (i.e., decoy molecules or miRNAs) (reviewed in [123]). Suppressors of RNAi are virus-encoded proteins that either inhibit the RNAi machinery (e.g., Dicer, Argonaute) or bind viral RNAs and protect them from Dicer-mediated cleavage or loading onto the RNA-induced silencing complex (RISC) (reviewed in [124,125,126]). The FHV-encoded B2 protein is a VSR that suppresses RNAi-mediated virus silencing by binding both long double-stranded RNAs (dsRNAs) and short interfering RNAs (siRNAs). Binding of FHV-B2 to long dsRNA, including viral replicative intermediates, protects them from cleavage, whereas high affinity binding of B2 to siRNAs prevents their incorporation into RISC, both of which result in suppression of the host RNAi response [113,127,128].

Similar to other insects, honey bee antiviral defense involves numerous processes and pathways, including autophagy, endocytosis, apoptosis, eicosanoid signaling, melanization, and the Toll and Imd (Immune Deficiency) pathways involving NF-κB (Nuclear Factor κB) homologues Dorsal and Relish, JAK/STAT (Janus Kinase/Signal Transducer and Activator of Transcription), JNK (c-Jun N-terminal kinase), MAPK (Mitogen-Activated Protein Kinases), and RNA interference (RNAi) pathways (reviewed in [23,129,130,131]). RNA interference is a sequence-specific post-transcriptional gene silencing mechanism that is induced upon recognition of long double-stranded RNA (dsRNA) (endogenous or virally derived) by the endonuclease Dicer. Dicer cleaves dsRNA into 21–22 bp double-stranded short interfering RNAs (siRNAs). Then, in a process mediated by heat shock proteins, a single strand, called the guide strand is retained by Argonaute-2, within the RNA-induced silencing complex (RISC) [132,133]. RISC surveils the cell, binds RNA complementary to the guide strand, and cleaves it [108,112,124,130,131,134,135,136,137,138,139]. In honey bees and bumble bees, dsRNA, a virus-associated molecular pattern, also induces a non-sequence-specific antiviral immune response that limits virus infections [107,108,109,140,141,142,143,144]. To date, investigations of bee antiviral defense have been performed primarily in pupae and adult bees [95,96,101,105,106,107,108,109,138,139,140,141,142,143,145,146,147,148,149,150] (reviewed in [23,129]). Further development and use of honey bee cell cultures will enable more mechanistic studies of bee virology and immunology.

There is only one immortalized honey bee cell line, AmE-711, which was established from honey bee embryonic tissue [9] (reviewed in [8]). This cell line was used to study the dynamics of several co-infecting honey-bee-infecting viruses, including sacbrood virus (SBV), deformed wing virus (DWV), Israeli acute paralysis virus (IAPV), black queen cell virus (BQCV), and Kashmir bee virus (KBV) [10]. In that study, AmE-711 cells were inoculated with a mixed virus preparation that included varying levels of SBV, DWV, IAPV, and BQCV. In inoculated cells, IAPV outcompeted SBV, DWV, and BQCV; however, when cells were infected with a different inoculum that contained KBV, SBV, IAPV, and BQCV, KBV outcompeted all other viruses, including IAPV [10]. While an important step towards the development of immortalized honey bee cell lines, AmE-711 has a long doubling time, is difficult to maintain in other labs, and it is persistently infected with DWV [10]. Therefore, the use of non-honey-bee cells lines, including the Lepidopteran hemocytic cell line (P1), to study honey-bee-infecting viruses, including DWV, have been explored and will likely serve as an important tool for investigating bee viruses at a cellular level [8,151]. While immortalized cell cultures provide a convenient and consistent tool, they often do not fully recapitulate in situ biology. For example, the immune response that AmE-711, a honey bee fibroblast-type cell line, mounts against viruses may not represent the immune response in other honey bee tissues or cell types (i.e., fat body, endothelium, or hemocytes).

Primary honey bee cell cultures derived from different tissues provide a useful alternative to immortalized cell lines for the study of basic honey bee virology and immunology (reviewed in [152]). There are several advantages and disadvantages associated with the use of either immortalized cell lines or primary cells. For example, primary cultures may harbor preexisting infections, while immortalized cells may be persistently infected; therefore, it is best to carry out pathogen testing to ensure that the results obtained are not impacted by other infections [153,154,155,156,157,158,159,160]. Beneficial features of tissue-derived primary cell cultures include that they can contain diverse cell types or be enriched for one cell type. In addition, they can be generated from pooled tissue samples to control for the inter-individual variability associated with whole-animal experiments. Primary cell cultures that represent multiple individuals may be particularly important for generating robust data that is representative of outbred honey bee colonies comprising individuals from numerous unique patrilines. Primary cell cultures established from whole pupae, with differentiated head, thorax, and abdomen body segments, likely include numerous adipocytes and epithelial cells as well as more specialized cells including neurons and muscle cells. These mixed cell cultures may be beneficial for investigating virus-tropism and intercellular signaling in response to virus infection. Similarly, the establishment and use of primary cell cultures from specific tissues or cell types facilitates a more precise mechanistic-level understanding of host–virus interactions [3,161,162]. Hemocytes are the primary insect immune cells that phagocytose or encapsulate pathogens. There are several subtypes of hemocytes, which are characterized morphologically, histochemically, and/or functionally. These include prohemocytes, granulocytes, plasmatocytes, spherulocytes, and oenocytoids, which may be further distinguished based on functional diversity [163]. Honey bee hemocytes also exhibit functional and morphological heterogeneity. The majority of circulating phagocytic cells in bee larvae are granulocytes, whereas plasmatocytes are more prevalent in adults [164,165]. There may be additional infection-induced cell types not yet described, like lamellocytes in *Drosophila melanogaster*, which are induced upon infestation by a parasitoid wasp [166]. In addition to their heterogeneity in phagocytic ability, honey bee hemocytes differ in their expression of hemolectin, which is associated with an encapsulation response of foreign bodies [167]. While recent studies in *Drosophila melanogaster* demonstrate that hemocytes play a role in systemic antiviral immune response, the potential for hemocytes to be infected by viruses or the extent to which they may contribute to honey bee antiviral immunity is not known [96,163,168,169,170,171].

To investigate honey bee antiviral defense mechanisms at the cellular level, we further developed the use of cultured primary cells, derived from either larvae or pupae, and demonstrated that these cells could be infected with a panel of viruses, including common honey bee infecting viruses (i.e., SBV and DWV) and a model virus (FHV). Specifically, hemocytes obtained from stage L4-L5 honey bee larvae supported infection and replication of SBV and FHV. Virus replication was verified via detection of the replicative intermediate of both viruses using negative-strand-specific reverse transcription (RT), followed by polymerase chain reaction (PCR), and using quantitative PCR (qPCR) to demonstrate increased virus abundance over an infection time course. Mixed cell populations derived from purple-eyed honey bee pupae supported replication of SBV, DWV, and FHV. Virus replication was verified via negative-strand-specific RT-PCR and qPCR. Peak virus abundance was observed at 96 h post-infection (hpi) for SBV and DWV, while virus abundance peaked at 72 hpi for FHV. Furthermore, the production of infectious virions in cultured honey bee pupal cells was demonstrated by determining that naïve cells became infected after the transfer DWV or FHV infected cell culture material. Initial characterization of the honey bee antiviral immune responses mounted against SBV, DWV, and FHV at the cellular level indicate varying virus-specific responses, including increased expression of *bee antiviral protein-1* (GenBank: MF116383) in SBV-infected pupal cells and increased expression of *argonaute-2* and *dicer-like* in FHV-infected hemocytes and pupal cells [107,172]. Additional studies utilizing primary honey bee cell cultures and multiple viruses will help elucidate virus-specific antiviral defense mechanisms.

## 2. Materials and Methods

### 2.1. Honey Bee Colonies

Packages (~1.5 kg of worker bees and a mated queen) of Carniolan honey bees (*Apis mellifera carnica*) were purchased from a commercial producer in Montana and installed in Langstroth hives at Montana State University, Bozeman, MT, USA. Colonies were maintained using standard apicultural practices, including bi-monthly *Varroa destructor* mite checks by the sugar roll method and treatment for mites with Mite Away Quick Strips (Nature’s Own Design Apiary Products, Frankford, ON, Canada) when mite infestation was evaluated to be greater than 3% (3 mites per 100 bees [173,174]).

### 2.2. Honey Bee Cell Cultures

#### 2.2.1. Hemocytes

Three days before experiments using primary honey bee hemocytes were performed, frames of brood with a lot of 4th and 5th instar larvae (L4–L5) were brought to the laboratory and maintained in a humidified incubator at 28 °C. Larvae that fell out of their cells were caught in a glass dish and collected in batch the next morning. After collection, larvae were surface-sterilized for 3 min in 0.6% hypochlorite (dilute bleach), followed by 3 min in 70% ethanol, and then briefly washed in sterile water for injection (Gibco) in a biological safety cabinet (Class II type A/B3, Nuaire). Larvae were placed into 200 μL of WH2 media (pH 6.4, 150 mL Schneider’s insect medium, SIGMA cat# S0146; 200 mL 0.06M L-Histidine, SIGMA cat# H5659; 50 mL heat-inactivated fetal bovine serum, Life Technologies cat# 16000044; 15 mL CMRL medium, Gibco cat# 11530-037; 5 mL Hank’s balanced salts, SIGMA cat# H9394; 2 mL insect media supplement, SIGMA cat# I7267), supplemented with 1× penicillin/streptomycin (ThermoFisher, cat# 15070063), 10 at a time [33,175]. Sterile 18-guage needles were used to wound larvae on the lateral cuticle of the abdomen. Each larva was wounded once on each side and exsanguinated directly into the media. Care was taken to minimize fat body release from the carcass to produce cultures with minimal adipocyte contamination (Supplemental Figure S1). Hemocytes were pooled in 15 mL conical tubes (Falcon, Corning) until enough were collected for each experiment. Hemocytes were then seeded in vacuum gas plasma treated polystyrene 48-well plates (Falcon, Corning). On average, six larvae provided enough hemocytes (~1 × 10^5^ cells per well) for five wells on a 48-well plate.

To test for virus association with carcasses versus hemocytes, an independent set of larvae was obtained from two different colonies, and sterile 18-guage needles were used to wound individual larva (*n* = 37) on the lateral cuticle of the abdomen. Each larva was wounded one on each side and exsanguinated. Hemolymph was directly collected by pipetting (approximately 40 μL). Hemolymph was diluted with 100 μL WH2 media; then, hemocytes were pelleted by centrifuging at 1000× *g* for 10 min. Supernatant was removed, and hemocytes were suspended in 100 μL WH2 media. Then, 800 μL of TRIzol^®^ reagent (Invitrogen) was added to the hemocytes, and they were stored at −80 °C until RNA extraction.

#### 2.2.2. Pupal Cells

Two days prior to honey bee pupal cell experiments, a brood frame containing purple-eyed pupae was brought into the lab. Fine-point, curved-tip forceps (Bioquip) were used to carefully remove the wax capping from pupae. Wide-tip, featherweight forceps (Bioquip) were used to gently grasp pupae between the eyes to remove them from the wax comb cells in which they develop. Pupae were incubated at 28 °C overnight in 12-well plates, and wounded (i.e., melanized) pupae were discarded. Primary cells were harvested from surface-sterilized honey bee pupae in a biosafety cabinet (Class II type A/B3, Nuaire). Surface sterilization was carried out in a sterile polystyrene petri dish (100 mm × 15 mm, Fisher), in which pupae were swirled in 0.6% hypochlorite solution (diluted bleach) for 3 min, 70% ethanol for 3 min, and briefly in sterile water for injection (Gibco). In groups of two, pupae were dissected into head, thorax, and abdomen segments in 2 mL WH2 medium in a 47 mm dish using sterile 18-gauge needles to vigorously disturb tissues and release cells into the medium [175]. The media containing the cells was then transferred to a 50 mL conical tube, and cells from individual pupa (~24 × 10^6^ cells) were pooled. Cell suspensions with approximately 10^6^ cells per 300 μL were plated into each well of a 48-well plate and incubated at room temperature overnight before infection.

### 2.3. Virus Preparation and Infection

#### 2.3.1. Sacbrood Virus

A stock of sacbrood virus was prepared from five symptomatic larvae, which were homogenized in 10 mM Tris (pH 7.5) using a sterile steel ball (5 mm) in a 2 mL microcentrifuge tube, using a Tissue Lyser II (Qiagen) at 30 Hz for 2 min. Cell debris was pelleted by centrifugation at 10,000× *g* for 10 min at 4 °C, and the clarified lysate was sequentially filtered through a 0.45 μm filter and then a 0.2 μm filter to remove small debris and microbes. To characterize this virus preparation, RNA was isolated from 100 μL using TRIzol^®^ reagent (as described below), and cDNA template was used for pathogen-specific PCR and qPCR. PCR confirmed the presence of SBV and the absence of other common honey bee viruses, including ABPV, BQCV, CBPV, DWV, IAPV, KBV, and LSVs (Supplemental Figure S11). Quantitative PCR was used to determine the relative abundance of SBV, using SBV RNA copy number as a proxy for virus abundance, while recognizing that in this crude virus preparation, RNA copies may represent both RNA genomes and transcripts. The SBV inoculum contained 5900 copies/ng RNA. Each well of a 48-well plate was inoculated with 2.5 × 10^6^ SBV RNA copies.

#### 2.3.2. Deformed Wing Virus

DWV inoculum was prepared according to standard methods [176]. In brief, white-eyed pupae were carefully removed from the brood comb and collected into a petri dish lined with filter paper dampened with sterile water. Microcapillary glass needles for injections were made by pulling borosilicate glass capillary tubes (100 mm long, 1 mL capacity, Kimble-Chase) with a coil temperature of 61 °C on the PC-10 Dual-Stage Glass Micropipette Puller (Narishige). Pupae were injected with DWV (3.41 × 10^7^ DWV RNA copies in 2 μL) between the 2nd and 3rd integuments of the abdomen using the borosilicate glass needle and a Harbo syringe (Honey bee Insemination Service). Pupae were incubated at 30 °C in a humid incubator over the course of infection and examined daily to ensure they were alive. Live pupae were harvested at 10 days post-injection, and individual pupae were homogenized in 1 mL PBS (pH 7.4) using a sterile steel ball (5 mm) and Tissue Lyser II (Qiagen) at 30 Hz for 2 min. Cell debris was pelleted by centrifugation at 10,000× *g* for 10 min at 4 °C. To characterize this virus preparation, RNA was isolated from 100 μL using TRIzol^®^ reagent (Invitrogen) (as described below), and cDNA template was used for pathogen-specific PCR and qPCR. PCR confirmed the presence of DWV and the absence of other common honey bee viruses, including ABPV, BQCV, CBPV, IAPV, KBV, LSVs, and SBV (Supplemental Figure S12). Quantitative PCR was used to determine the relative abundance of DWV, using virus RNA copy number as a proxy for virus abundance, while recognizing that in this crude virus preparation, RNA copies may represent both RNA genomes and transcripts. The DWV inoculum contained 5.48 × 10^7^ DWV RNA copies/μL. For DWV infection studies, approximately 10^6^ pupal cells in 300 μL (per well in a 48-well plate) were inoculated with 4.3 × 10^6^ DWV RNA copies.

#### 2.3.3. Flock House Virus

FHV was propagated in *Drosophila melanogaster* Schneider 2 (S2) cells (Invitrogen), an immortalized cell line originally derived from *D. melanogaster* embryos. S2 cells were grown in Schneider’s *Drosophila* medium supplemented with 10% heat-inactivated fetal bovine serum (Life Technologies) and 1% penicillin-streptomycin. A sterile T75 flask was seeded with 4 × 10^7^ S2 cells/mL and infected with FHV at a multiplicity of infection of 1 pfu/cell, as provided by Dr. Anette Schneemann (The Scripps Research Institute, La Jolla, CA, USA). The virus stock was quantified via plaque assay by the Schneemann lab using previously described protocols [177]. The S2 cells infected using this aliquot were incubated at 28 °C for 48 h, lysed by addition of 10% (*v*/*v*) Nonidet P-40, and incubated on ice for 10 min with periodic swirling [178]. Cell debris was pelleted at 13,800× *g* for 10 min at 4 °C, and the clarified supernatant was transferred to a fresh tube. Using an ultracentrifuge (Beckman Coulter), virus was pelleted through 1 mL volume of 30% (*wt*/*wt*) sucrose in 50 mM HEPES (pH 7) at 40,000 rpm for 2.5 h at 11 °C in a SW41 Ti swinging-bucket rotor. The resulting pellets were resuspended in 0.5 mL buffer (HEPES 50 mM, pH 7), and any remaining insoluble material was removed by centrifugation at 10,000 rpm for 10 min. The clarified supernatant was layered on a continuous sucrose gradient (40%, 35%, 30%, 25%, 20%, 15%, and 10% (*wt*/*wt*)) and centrifuged at 40,000 rpm for 1.5 h at 11 °C to sediment the virus between 25–35% sucrose gradients. RNA was isolated from 100 µL virus preparations (as described below), and FHV abundance was quantified by qPCR, with the copy number based on a standard curve. For the infection experiments described herein, hemocytes were infected with 1 × 10^6^ FHV RNA copies, and pupal cells were infected with 2 × 10^8^ FHV RNA copies. FHV infection experiments were monitored over a time course from 0 h to 96 h post-infection.

### 2.4. Transfer of Virus Infection from Infected to Naïve Primary Honey Bee Pupal Cells

Small amounts of virus-infected cell culture, including cells and culture supernatant, from initial infections were transferred to naïve pupal cells, seeded in 48-well plates as described above. Primary purple-eyed honey bee pupal cells were seeded at approximately 10^6^ cells per 300 μL media and incubated at room temperature overnight before infection. Each well was infected with 10 µL of mock- or virus-infected pupal cell culture. The resulting infected cells were incubated at room temperature for 0 h, 48 h, and 72 h post-infection.

### 2.5. RNA Isolation

RNA was isolated from cultured adherent hemocytes or non-adherent pupal cells/cell culture media at indicated time points using TRIzol^®^ reagent (Invitrogen) according to manufacturer’s instructions. In brief, cell culture medium from each well (300 µL) was collected into a microcentrifuge tube. Then, 300 μL of TRIzol^®^ reagent (Invitrogen) was added to each well, incubated for 1 min, and then combined with the corresponding cell culture media sample. Samples were stored at −80 °C until RNA extraction. To complete RNA isolation, an additional 450 μL of TRIzol was added to each sample, which was vortexed and incubated at room temperature for 5 min. Then, 160 μL of chloroform was added, and samples were shaken by hand (15 s) and incubated at room temperature (2 min). Samples were centrifuged at 12,000× *g* for 15 min, and the aqueous phase was transferred to a clean microcentrifuge tube. An equal volume of isopropanol (~550 μL) and 20 μg of glycogen (Thermo Scientific) were added to the aqueous phase, which was then mixed by inverting. RNA was precipitated after overnight incubation (~18 h) at −20 °C by centrifugation at 4 °C for 15 min at 14,000× *g*. Supernatants were carefully removed by pipetting, and each pellet was washed twice with 75% ethanol. Final pellets were air-dried at room temperature until visually dry (~20 min). The pellet was suspended in 30 μL nuclease-free water; then, 3 μL 3M sodium acetate (pH 5.5) and 120 μL ethanol were added, and RNA was precipitated overnight at −20 °C. Next, samples were centrifuged at 14,000× *g* for 15 min, and supernatants were carefully removed by pipetting. Each pellet was washed twice with 75% ethanol. Finally, RNA pellets were air-dried at room temperature until visually dry (~20 min) and then dissolved in 20 μL nuclease-free water. RNA concentration was measured using a NanoDrop 2000 spectrophotometer (Thermo Fisher).

To extract RNA from larval carcasses, 300 μL of deionized H_2_O was added to the exsanguinated carcass. The carcass was homogenized with a sterile steel BB using a TissueLyzer (Qiagen) at 30 Hz for 2 min. Then, 800 μL TRIzol^®^ reagent (Invitrogen) was added to the lysate and vortexed and incubated at room temperature for 5 min; 200 μL of chloroform was then added. The tubes were shaken by hand for 15 s and incubated at room temperature for 2 min. The samples were then centrifuged for 15 min at 4 °C at 12,000× *g*. The aqueous phase was transferred to a clean microcentrifuge tube (approximately 800 μL), one volume of isopropanol was added, and tubes were mixed by inverting. Samples were incubated at −20 °C for one hour and then centrifuged for 10 min at 4 °C at 12,000× *g.* The supernatant was decanted, and the pellet was washed by adding 500 μL 75% ethanol and centrifuging for 5 min (7500× *g*) at 4 °C. The supernatant was decanted, and pellets were air-dried at room temperature. Pellets were suspended in 100 μL of dH_2_O.

### 2.6. Reverse Transcription/cDNA Synthesis

Reverse transcription (RT) reactions to produce complementary DNA (cDNA) were performed by incubating 100–250 ng total RNA for cultured cell experiments, 200 units M-MLV reverse-transcriptase (Promega), and 500 ng random hexamer primers (IDT) for 2 h at 37 °C, according to the manufacturer’s instructions. For larvae/hemocyte assays, 2000 and 1000 ng total RNA, respectively, were used for RT reactions.

### 2.7. Negative Strand-Specific RT-PCR

Virus-treated samples (with SBV, DWV, or FHV) were analyzed for the presence of the negative-strand RNA of the viral genome (i.e., the replicative intermediate form) using strand-specific PCR [88,179,180]. Reverse transcription/cDNA synthesis reactions were performed with M-MLV (Promega) according to the manufacturer’s instructions using negative strand-specific primers tagged with an additional 21 nt of sequence at their 5′ end (Supplemental Table S1). The tag sequence (5′GGCCGTCATGGTGGCGAATAA3′) shares no homology with the viruses used in this study nor with the honey bee genome. Reactions were carried out by incubating 100–250 ng RNA and primers (10 pmol specific or 500 ng hexamers) with 200 units M-MLV reverse-transcriptase (Promega) according to the manufacturer’s instructions. Reverse transcription reactions were incubated for 2 at 37 °C. Unincorporated primers present in the RT reactions were digested with 2 units exonuclease I (Life Technologies) per reaction at 37 °C for 30 min, followed by heat inactivation at 85 °C for 5 min. To detect negative-strand-derived cDNA products, 5 μL of cDNA template in 12.5 μL aqueous buffer containing 10 pmol of each of a tag-specific forward primer (TAGS) and virus-specific reverse primer (Table S1) was amplified with 0.5 μL (5 U/μL) ChoiceTaq polymerase (Thomas Scientific) using the following cycling conditions: 95 °C for 5 min; 95 °C for 30 s, 55 °C for 30 s, 72 °C for 45 s, 35 cycles; final elongation 72 °C for 5 min, hold at 4 °C. Negative control PCR reactions were performed using template generated from RNA-containing RT reactions that were incubated in the absence of RT enzyme or using cDNA template that was generated using random-hexamer primers and only including the reverse primer in the PCR. Positive controls included PCR using virus-specific qPCR primers (Supplemental Table S1). Viral and other long single-stranded RNA molecules often exhibit secondary structures, which serve to prime reverse transcription reactions in the absence of exogenous primers (i.e., self-priming). To test for self-priming, RT reactions were carried out in the absence of primers, followed by PCR with virus-specific qPCR primers (See Supplemental Table S1 for full list of controls).

### 2.8. Polymerase Chain Reaction (PCR)

PCR was performed according to standard methods [33,63,64,97]. In brief, 2 μL cDNA template in 12.5 μL aqueous buffer containing 10 pmol of each forward and reverse primer (Table S1) was amplified with 0.5 μL (5 U/μL) ChoiceTaq polymerase (Thomas Scientific) according to the manufacturer’s instructions using the following cycling conditions: 95 °C for 5 min; 95 °C for 30 s, 55 °C for 30 s, 72 °C for 45 s, 35 cycles; final elongation 72 °C for 5 min, hold at 4 °C. PCR products were visualized by 1.5% agarose gel electrophoresis and stained with SYBRsafe (Invitrogen). Positive and negative control reactions were included for all analyses and exhibited the expected results. Select products were purified with the Qiaquick PCR Purification Kit (Qiagen), quantified using a NanoDrop spectrophotometer (Fisher), and Sanger sequenced.

### 2.9. Quantitative PCR

Quantitative PCR (qPCR) was used to quantify the viral RNA (i.e., genome and transcript) abundance in the samples and assess the relative abundances of honey bee encoded transcripts (i.e., a housekeeping gene, *rpl8*), and three immune genes (i.e., *ago2*, *dcr-like*, and *bap1/mf116383*) using the primer sets listed in Supplemental Table S1. All qPCR reactions were performed in triplicate using 2 μL of cDNA template and a no-template negative control was run in triplicate for each qPCR reaction. Each 20 μL reaction contained 1× Denville ChoiceTaq Mastermix, 0.4 μM each forward and reverse primer, 1× SYBR Green (Life Technologies), and 3 mM MgCl_2_. Reactions were carried out in 96-well plates using a CFX Connect Real-Time instrument using Maestro software (Bio-Rad) with the following thermo-profile: initial denaturation at 95 °C for one minute; followed by 40 cycles of 95 °C for 10 s, 58 °C for 20 s, and 72 °C for 15 s, with a final melt curve analysis at 65 °C for 5 s to 95 °C. Positive and negative control reactions were included for all qPCR analyses and exhibited the expected results. Viral RNA copies were estimated using a plasmid DNA standard for SBV, DWV, and FHV, with a detection range of 10^3^ to 10^9^ to create a linear standard curve used for copy number interpolation. The linear equation for the plasmid standard for FHV was Ct = −3.240x + 40.138 (R^2^ = 0.980, efficiency = 103%), determined by a previously described method [181]. Similarly, the linear equation for DWV was Ct = −3.5627x + 39.091 (R² = 0.9973, efficiency = 90.5%). The linear equation for the SBV standard curve was Ct = −3.39x + 37.42 (R^2^ = 0.994, efficiency = 97.2%). The relative expression of host genes was determined by a ranked ΔΔCt method in which the ΔCt was calculated by subtracting the *rpl8* Ct value from the Ct of the gene of interest. Then, the within-group ΔCt values were ranked from highest to lowest, and the relevant corresponding control ΔCt value was subtracted from the treatment group ΔCt to obtain the ΔΔCt. The fold-change in cDNA abundance was calculated by the equation 2^−ΔΔCt^. See Supplemental Tables S2–S12 for qPCR data presented in the figures.

### 2.10. Microscopy

To prepare hemocytes for imaging, they were isolated as above and seeded into #1.5 German cover glass chamber slides (Thermo Fisher Scientific) in WH2 media. Live-cell and fixed-cell images were taken on a Nikon Ti-Eclipse (Nikon Instruments) inverted microscope equipped with a SpectraX LED (Lumencor) excitation module and fast-switching emission filter wheels (Prior Scientific). Brightfield images were captured using a Plan Fluor 20 phase contrast (Ph) objective and an iXon 896 EM-CCD (Andor Technology Ltd.) camera using NIS Elements software. To visualize cells with an intact nucleus, the cell culture media was removed, and the cells were washed with PBS and then incubated in 4% paraformaldehyde in PBS for 10 min at room temperature. The paraformaldehyde containing PBS was removed, and cells were washed two times with PBS. Hoechst stain (Hoechst 33258, Invitrogen) prepared to 30 mg/mL in PBS was further diluted 1:5000 in PBS to create a working solution, which was then overlaid on the cells. Then, fluorescent images were captured using paired excitation/emission filters and dichroic mirrors for DAPI (Chroma Technology Corp).

### 2.11. Statistical Analysis

All data were analyzed using R v4.0.2 in RStudio V1.3.1073. Unless otherwise stated, comparisons for virus abundance were evaluated using the *DunnettTest* function in the DescTools R stats package to perform the post hoc pairwise multiple comparisons to a control [182]. Relative gene expression differences were evaluated using a one-sided Wilcoxon [183]. For each analysis, comparisons were considered significant if *p* < 0.05.

## 3. Results and Discussion

### 3.1. Sacbrood Virus (SBV) Replicates in Primary Honey Bee Hemocytes

**Figure 1:**
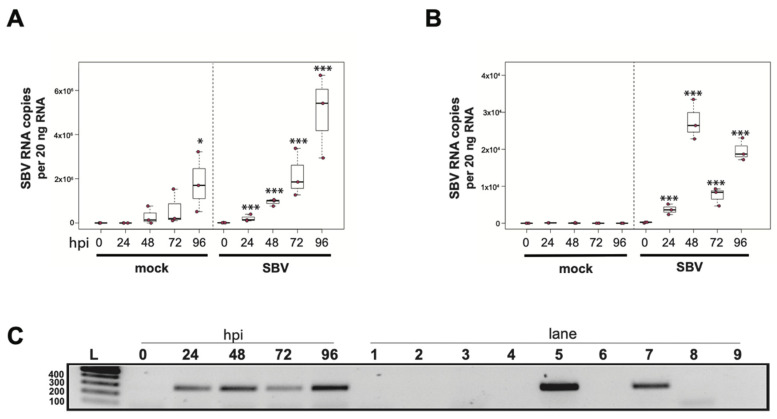
Sacbrood virus (SBV) naturally infects hemocytes and replicates in primary honey bee hemocyte cell cultures. SBV abundance was assessed in mock- or SBV-treated larval hemocytes by qPCR over a time course (i.e., 0 hpi, 24 hpi, 48 hpi, 72 hpi, and 96 hpi). (**A**) Cells isolated from asymptomatic larvae and treated with PBS (pH 7.4) had a 501× increase in mean SBV abundance by 96 hpi relative to 0 hpi (*p* = 0.042), indicating that the hemocytes in this experimental replicate had a pre-existing SBV infection and that hemocytes are a natural site of infection for SBV. The addition of filtered larval lysate containing 2.5 × 10^6^ SBV RNA copies resulted in a 13× increase by 24 hpi relative to 0 hpi (*p* < 0.001) and a peak fold increase of 355× by 96 hpi (*p* < 0.0001). (**B**) In a second replicate of the experiment, mock-infected cells remained uninfected over the course of the experiment. The addition of filtered larval lysate containing 2.5 × 10^6^ SBV RNA copies resulted in a peak fold increase of 182× (*p* < 0.0001) at 48 hpi, with a decrease at 72 hpi (49× relative to 0 hpi, *p* < 0.0001) and a subsequent rise again at 96 hpi (132× relative to 0 hpi, *p* < 0.0001). Raw data are included in Supplemental Table S2. All differences in means relative to 0 hpi were assessed by a Dunnett’s test. Significance levels: * *p* < 0.05; *** *p* < 0.0005. (**C**) To confirm that SBV was productive in infecting larval hemocytes, the presence of the negative strand (a replicative intermediate) was assessed by negative-strand-specific reverse transcription (RT) followed by PCR. Negative strand was detected at all time points after 0 hpi. Additional control reactions were performed using pooled RNA from time points 24–96 hpi (Lanes labeled 1–9). Specifically, RNA isolated from SBV containing cell lysate was reverse transcribed with the primer listed below, treated with Exonuclease I to remove excess primer, and amplified using the PCR primers listed for each lane. (L) Molecular weight ladder. (0, 24, 48, 96 hpi)—RT with random hexamers, PCR with SBV-221-240-For and SBV-478-497-Rev. (1) Negative control: No RT in the presence of (SBV-TAGS-F), PCR with TAGS and SBV-478-497-Rev. (2) Negative control: RT in the presence of (SBV-TAGS-F), PCR with only the SBV-478-497-Rev primer. (3) Negative control: RT with random hexamer primer, PCR with TAGS and SBV-478-497-Rev. (4) Negative control: No RT enzyme in the presence of random hexamers, PCR with SBV-221-240-For and SBV-478-497-Rev. (5) Positive control: RT with random hexamers, PCR with SBV-221-240-For and SBV-478-497-Rev. (6) Negative control: RT with random hexamers, PCR with only SBV-478-497-Rev primer. (7) Self-priming: RT without a primer, PCR with SBV-221-240-For and SBV-478-497-Rev. (8) Negative control: No template, no RT, PCR with TAGS and SBV-478-497-Rev. (9) Negative control: No template, no RT, PCR with SBV-221-240-For and SBV-478-497-Rev. Additional details provided in Supplemental Table S25. When reverse transcription was performed without primer, SBV cDNA was detectable (lane 7), indicating that the secondary structure in the RNA genome may serve as a primer for cDNA synthesis.

To determine whether sacbrood virus (SBV) infects hemocytes, the primary immune cell in honey bees, approximately 1 × 10^5^ larval (L4–L5) hemocytes were seeded into wells of a 48-well tissue culture plate with relatively few contaminating adipocytes and treated with either phosphate buffered saline (PBS) or SBV (i.e., bee larval lysate containing 2.5 × 10^6^ SBV RNA copies) (Supplemental Figure S1). Cells were harvested at distinct time points post-infection (i.e., from 0 hours post-infection (hpi) to 96 hpi) and virus abundance, using RNA copy number as a proxy, was determined by qPCR. In one experimental replicate, the primary honey bee hemocyte culture had a pre-existing SBV infection, which exhibited a 501× increase in SBV by 96 h post-treatment (*p* = 0.042) (Figure 1A). This result indicates that hemocytes are a natural site of SBV infection and that SBV virus infections can persist in cells grown in culture. The addition of SBV increased the infection level by 13× over the initial levels by 24 hpi (*p* < 0.001), and it rose to 69.2× by 48 hpi (*p* < 0.001), to 147.9× by 72 hpi (*p* < 0.0001), and peaked at 354.8× at 96 hpi (*p* < 0.0001). In a second biological replicate of this experiment, confounding SBV infection was not detected (Figure 1B). In inoculated hemocytes, there was a 24× increase from 0 hpi to 24 hpi (*p* = 0.0001), and by 48 hpi SBV had increased 182× (*p* < 0.0001) and reached peak infection. There was a decrease in SBV relative to 48 hpi at 72 hpi (i.e., 49× greater virus levels than at 0 hpi, *p* < 0.0001), with a subsequent rise at 96 hpi (i.e., 132× increase over 0 hpi, *p* < 0.0001). Although it is unclear why there was a reduction in viral abundance at 72 hpi, it could have been due to a decrease in the number of permissive hemocytes in the culture; by 96 hpi, cell division and/or differentiation may have occurred and resulted in additional susceptible host cells.

To further validate that the increase in SBV over the infection time course was due to active virus replication in honey bee hemocytes, we carried out strand-specific RT-PCR. The SBV genome is positive-sense single-stranded RNA (+ssRNA). During virus replication, a negative strand template is produced [88,179,180,184]. The negative strand of SBV was not detected in the inoculum (i.e., at 0 hpi), but was detected at all other time points (Figure 1C). Together these data indicate that not only does SBV actively replicate in hemocytes, but hemocytes also serve as a natural site of virus infection. Furthermore, virus testing of honey bee larvae (i.e., corresponding carcass and hemocyte samples) indicate that hemocytes are natural sites of infection for black queen cell virus (BQCV) (Supplemental Table S24). While, in general, hemocytes had low incidence of infection, isolated larval hemocytes were positive for BQCV in 9.1% of cases where the larval carcass was also BQCV-positive (Supplemental Table S24). Together, these data suggest hemocytes might be a site of infection for multiple viruses. These results are in line with reports of virus infections in other invertebrates, including persistent infection of hemocytes by hepatitis A virus (HAV) and murine norovirus (MNV) in oysters (*Crassostrea virginica*) [185]. In addition, white spot syndrome virus (WSSV) infects subpopulations of shrimp hemocytes [186]. Results from honey bees, oysters, and shrimp differ from the observation that hemocytes of Sindbis-virus-infected *Drosophila melanogaster* remain uninfected [170]. As described above, hemocytes consist of several subpopulations. Future studies aimed at identifying the hemocyte subpopulations that are permissive to specific viruses will provide insight into the role of these important immune cells in virus dissemination and antiviral defense. Understanding how viruses interact with hemocytes and how they might frustrate the cellular immune function may aid in the long-term goal of developing therapeutics and technologies that mitigate the negative impacts of viruses on bees.

To our knowledge, this is the first quantitative evidence that SBV productively infects honey bee hemocytes. A previous study used transmission electron microscopy to visualize SBV virions within larval and adult hemocytes [168]. While those images indicated that SBV may replicate in hemocytes, they could have also been explained by phagocytosis of virions [168]. The observation that honey bee hemocytes are naturally infected by SBV has important implications for viral pathogenesis. While we did not observe any cytopathic effects (CPEs), it is possible the cells would have shown CPEs if the experiments were carried out for a longer time period. Additionally, the immune function of hemocytes may be dysregulated by viral infection, since viruses subvert cellular processes and disrupt cellular homeostasis [187,188]. For example, DWV suppresses larval melanization in response to wounding, suggesting an interaction between DWV and hemocytes [96]. This is further supported by gene expression studies of DWV-infected larvae, which exhibited reduced expression of *Amel*\102, a gene involved in melanization and encapsulation [96].

In addition to hosting viral infection, honey bee hemocytes are likely instrumental in antiviral immune responses, as they are in *Drosophila melanogaster*. Direct evidence for an antiviral role for hemocytes in fruit flies includes the results of in vivo experiments in which hemocytes were either saturated via injection of latex beads or genetically depleted [189]. Flies with reduced hemocyte function that were infected with either Cricket paralysis virus (CrPV), FHV, or vesicular stomatitis virus (VSV) had increased viral abundance and higher mortality than flies with phagocytic hemocytes. In contrast, reduced hemocyte function had no impact on Drosophila C virus (DCV), Sindbis virus (SINV), and Invertebrate iridescent virus 6 (IIV6) infections in flies [189]. These results also indicated that the relative importance of hemocytes in host antiviral defense will vary for specific host–virus pairs and may also depend on additional factors (e.g., co-infection, nutritional status, age). Hemocytes are required for systemic adaptive RNAi response in flies, which limits virus infection [170,171]. This finding provides strong support for an antiviral role of hemocytes against some viruses in fruit flies, which suggests that hemocytes likely serve an antiviral role in insects in general.

While the function of hemocytes in honey bee antiviral response requires further investigation, there is limited indirect evidence of their antiviral role. Specifically, genes involved in migration, phagocytosis, and expression immunoglobulin domain encoding, were expressed at greater levels in honey bees infected with a model virus, SINV, relative to mock-infected bees [107]. Additionally, IAPV infection resulted in increased expression of genes involved in cell projection, cellular organization, autophagic cell death, microtubule-based movement, and phagocytosis [101,190]. Our observation that viruses replicate in cultured hemocytes will facilitate future studies aimed at elucidating their role in antiviral defense, including further investigation of the genes and processes highlighted by transcriptional level studies.

### 3.2. Sacbrood Virus (SBV) Replicates in Primary Honey Bee Pupal Cell Cultures

**Figure 2:**
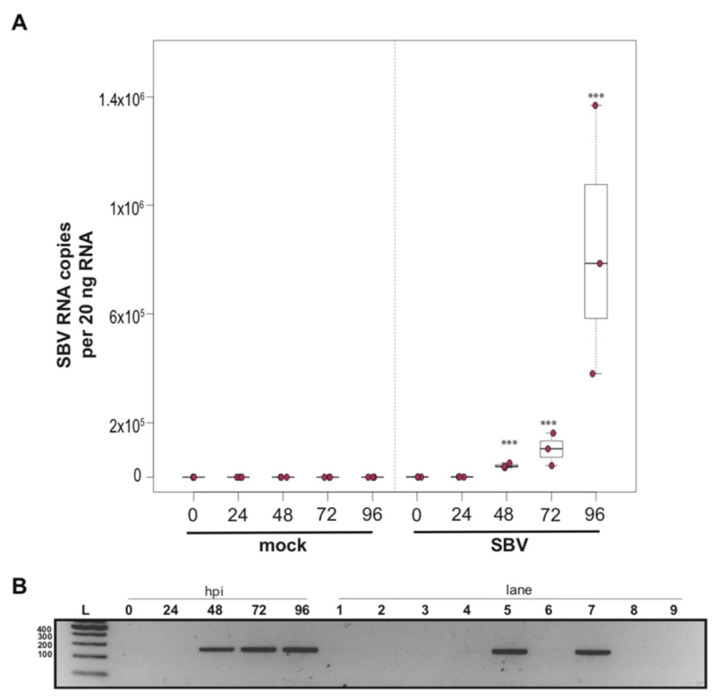
Sacbrood virus (SBV) replicates in primary honey bee pupal cell cultures. (**A**) SBV abundance was assessed in mock- or SBV-treated pupal cells by qPCR over a time course (i.e., 0 hpi, 24 hpi, 48 hpi, 72 hpi, and 96 hpi). A representative replicate of SBV-infection in pupal cells is plotted. The addition of larval lysate to pupal cells resulted in a 59× fold increase relative to 0 hpi by 48 hpi (*p* < 0.0001), with subsequent increases at 72 hpi (126×, *p* < 0.0001) and 96 hpi (1047×, *p* < 0.0001). Raw data are included in Supplemental Table S3. See Supplemental Figure S2 for additional replicates. All differences in means relative to 0 hpi were assessed by a Dunnett’s test. Significance levels: *** *p* < 0.0005. (**B**) To confirm that SBV was productive in infecting larval hemocytes, the presence of the negative strand (a replicative intermediate) was assessed by negative strand-specific reverse transcription (RT) followed by PCR. Negative strand was detected at all time points after 24 hpi. Additional control reactions were performed using pooled RNA from time points 24–96 hpi (Lanes labeled 1–9). Specifically, RNA isolated from SBV containing cell lysate was reverse-transcribed with the primer listed below, treated with Exonuclease I to remove excess primer, and amplified using the PCR primers listed for each lane. (L) Molecular weight ladder. (0, 24, 48, 96 hpi)—RT with random hexamers, PCR with SBV-221-240-For and SBV-478-497-Rev. (1) Negative control: No RT in the presence of (SBV-TAGS-F), PCR with TAGS and SBV-478-497-Rev. (2) Negative control: RT in the presence of (SBV-TAGS-F), PCR with only the SBV-478-497-Rev primer. (3) Negative control: RT with random hexamer primer, PCR with TAGS and SBV-478-497-Rev. (4) Negative control: No RT enzyme in the presence of random hexamers, PCR with SBV-221-240-For and SBV-478-497-Rev. (5) Positive control: RT with random hexamers, PCR with SBV-221-240-For and SBV-478-497-Rev. (6) Negative control: RT with random hexamers, PCR with only SBV-478-497-Rev primer. (7) Self-priming: RT without a primer, PCR with SBV-221-240-For and SBV-478-497-Rev. (8) Negative control: No template, no RT, PCR with TAGS and SBV-478-497-Rev. (9) Negative control: No template, no RT, PCR with SBV-221-240-For and SBV-478-497-Rev. Additional details provided in Supplemental Table S25.

To determine whether a mixed cell culture derived from purple-eyed pupae supported SBV infection, approximately 1 × 10^6^ cells were treated with either PBS or SBV (i.e., bee larval lysate containing 2.5 × 10^6^ SBV RNA copies). Cells were harvested at distinct time points post-infection, and virus abundance was assessed by qPCR. Three independent experiments were performed (Supplemental Figure S2). In replicate one, SBV abundance was 7.8× higher by 48 hpi than the levels after 0 hpi (*p* < 0.0001). SBV abundance remained high at 72 hpi (12×, *p* < 0.0001) and 96 hpi (11.4×, *p* < 0.0001) (Supplemental Figure S2A). Likewise, in the second experimental replicate, SBV abundance was higher by 48 hpi (59×, *p* < 0.0001), remained high at 72 hpi (126×, *p* < 0.001), and peaked at 96 hpi (1047×, *p* < 0.0001) (Figure 2A and Figure S2B). While SBV levels at 24 hpi were close to the levels of the 0 hpi time points in the first two biological replicates, they were significantly lower in replicate three (i.e., 0.017×, *p* < 0.0001). Like the other two replicates, SBV levels increased by 48 hpi (2.2×, *p* = 0.0012), remained high at 72 hpi (14×, *p* = 0.00013), and peaked at 96 hpi (72×, *p* < 0.0001) (Supplemental Figure S2C). The overall increase in SBV levels for replicate three was 1,817× (i.e., from 24 hpi to 96 hpi (*p* < 0.0001)), which was similar to replicate two, which had an overall 1047× increase, whereas the replication and relative increase in abundance for replicate one was lower (i.e., only 72×) (Supplemental Figure S2A). To further verify that SBV productively infected cultured honey bee pupal cells, we used strand-specific RT-PCR to assay for the negative strand (i.e., replicative intermediate form of SBV). The SBV-negative strand was detected at three time points post-infection, which corresponded to peak virus abundances (i.e., 48, 72, and 96 hpi) (Figure 2B). Together, these data demonstrate that primary honey bee pupal cell cultures are permissive to SBV infection. Furthermore, since the fold increase of SBV in honey bee pupal cells was greater than in hemocytes, we hypothesize that cell types present in pupal cultures, including endothelial cells and fibroblasts, may be more suitable hosts for SBV compared to immune cells.

While the primary concern of SBV infection of honey bee colonies is larval mortality, SBV also reduces adult lifespan. SBV infections in pupae may inhibit the melanization pathway by causing reduced expression of *prophenoloxidase activating enzyme* (*PPAE*) and increased expression of a putative serpin protein encoding gene (GB48820) [59,145]. Pupae with a suppressed melanization response may be more susceptible to infection after wounding by *Varroa destructor* mites. It is not known whether SBV-infected pupae can clear infections prior to eclosion, and in turn if SBV-infected pupae can develop into healthy adults. Therefore, tools to study the impact of SBV and the molecular mechanisms of antiviral defense at all life stages are needed and include the SBV-permissive cultured primary cells described herein.

### 3.3. Deformed Wing Virus Replicates in Primary Honey Bee Pupal Cells

**Figure 3:**
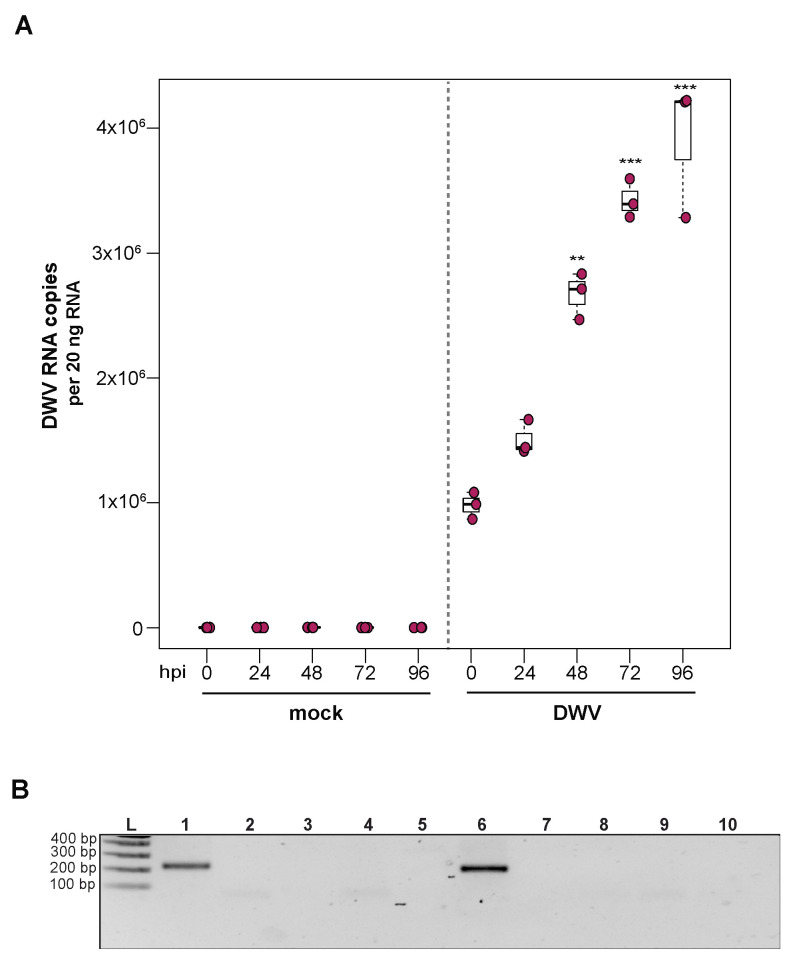
Deformed wing virus (DWV) replicates in primary honey bee pupal cell cultures. DWV abundance was assessed in mock- or DWV-infected pupal cells via qPCR over a time course (i.e., 0 hpi, 24 hpi, 48 hpi, 72 hpi, and 96 hpi). (**A**) Pupal cells infected with 4.3 × 10^6^ DWV RNA copies had a 2.7× (*p* < 0.001) and 3.5× (*p* < 0.001) increase in mean DWV abundance at 48 hpi and 72 hpi, respectively, relative to 0 hpi. At 96 hpi, mean DWV abundance was 4× higher relative to 0 hpi (*p* < 0.001). Figure 3 includes results from one representative biological replicate. The data for additional replicates are presented in Supplemental Figure S3. All differences in means relative to 0 hpi were assessed by a Dunnett’s test. Raw data are included in Supplemental Table S4. Significance levels: ** *p* < 0.005; *** *p* < 0.0005. (**B**) To verify DWV replication in purple-eyed pupal cells, negative-strand-specific RT-PCR was performed, and the replicative intermediate form (i.e., negative strand) of DWV was detected at 72 hpi. Additional control reactions were performed using pooled RNA from time points 24–96 hpi (Lanes labeled 2–9). Specifically, RNA isolated DWV containing cell lysate was reverse-transcribed with the primer listed below, treated with Exonuclease I to remove excess primer, and amplified using the PCR primers listed for each lane. (L) Molecular weight ladder. (1) 72 hpi RNA template—RT with DWV-TAGS-F, PCR with TAGS and DWV-Rev. (2) Negative control: No RT in the presence of (DWV-TAGS-F), PCR with TAGS and DWV-Rev. (3) Negative control: RT in the presence of (DWV-TAGS-F), PCR with only the DWV-Rev primer. (4) Negative control: RT with random hexamer primer, PCR with TAGS and DWV-Rev. (5) Negative control: No RT enzyme in the presence of random hexamers, PCR with DWV-F and DWV-Rev. (6) Positive control: RT with random hexamers, PCR with DWV-F and DWV-Rev. (7) Negative control: RT with random hexamers, PCR with only DWV-Rev primer. (8) Self-priming: RT without a primer, PCR with DWV-F and DWV-Rev (9) Negative control: No template, no RT, PCR with TAGS and DWV-Rev. (10) Negative control: No template, no RT, PCR with DWV-F and DWV-Rev. Additional details provided in Supplemental Table S25.

To investigate the utility of primary honey bee pupal cells to support DWV infection, approximately 1 × 10^6^ purple-eyed pupal cells were incubated with 4.3 × 10^6^ DWV RNA copies. Cells were harvested at distinct time points post-infection (i.e., from 0 hpi to 96 hpi), and virus abundance, using RNA copy number as a proxy, was determined by qPCR. Data from two independent experiments demonstrated that DWV abundance increased from 0 hpi to 96 hpi (Supplemental Figure S3). In replicate one, DWV levels remained unchanged for the first 24 h and were 2.7× greater at 48 hpi (*p* = 0.002). DWV abundance increased 3.5× and 4× at 72 hpi (*p* < 0.001) and 96 hpi (*p* < 0.001), respectively, with peak DWV levels at 96 hpi relative to 0 hpi (Figure 3A). In replicate two, DWV levels remained similar to inoculum levels through 48 hpi but were 3.2× higher at 72 hpi (*p* = 0.001) and 6.6× higher at 96 hpi, relative to 0 hpi (*p* = 0.003) (Supplemental Figure S3). To verify DWV replication in primary cultures of purple-eyed honey bee pupal cells, negative-strand specific PCR was performed, and the replicative intermediate form (i.e., negative strand) of DWV was detected at 72 hpi (Figure 3B). Together, DWV abundance and negative-strand data confirm that primary cultures of honey bee pupal cells support DWV replication.

The ability to infect primary honey bee cells with DWV opens new avenues for studying a pathogen that has co-evolved with honey bees. Primary cell cultures may also facilitate the propagation of virus stocks that are free of confounding viruses. Experiments in cultured honey bee cells also provide an opportunity to study the mechanistic details of DWV infections and virus–host dynamics in a system that complements experiments carried out in larvae, pupae, and adult honey bees. Recent efforts have focused on exploring the ability of alternative host cell lines to support honey bee virus infections; for example, the Lepidopteran hemocyte-derived cell line (P1) has been shown to support DWV infection [151]. While exploring the ability of alternative cell lines to support honey bee virus infections is an attractive approach, the immune response elicited by these alternative host cells may or may not completely recapitulate the immune response elicited by honey bees. Therefore, the potential to infect primary honey bee cells with naturally infecting honey bee viruses, including DWV, will be a powerful tool to explore the natural host–virus interactions at the cellular level.

### 3.4. Flock House Virus Replicates in Primary Honey Bee Hemocytes

**Figure 4:**
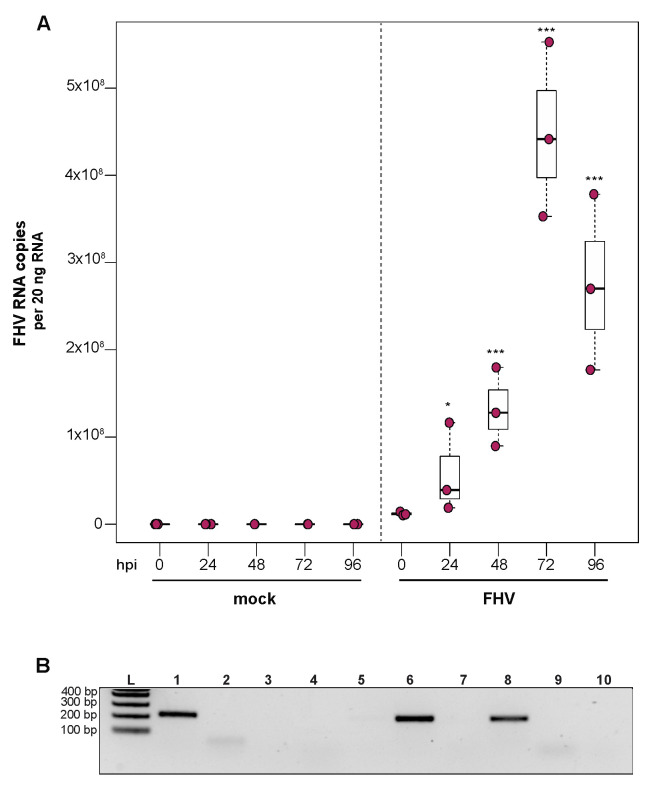
Flock House virus (FHV) replicates in primary honey bee hemocytes. FHV abundance in mock- or FHV-infected (1 × 10^6^ FHV RNA copies) hemocytes was assessed over time (i.e., 0 hpi, 24 hpi, 48 hpi, 72 hpi, and 96 hpi). (**A**) FHV abundance was higher at 24 hpi (3.7×, *p* = 0.026), 48 hpi (10.7×, *p* < 0.001), and 72 hpi (37×, *p* < 0.001), relative to 0 hpi. FHV abundance reduced from 72 hpi to 96 hpi (*p* = 0.034) but was significantly higher (*p* < 0.001) relative to 0 hpi. Raw data are included in Supplemental Table S5. Figure 4 includes results from one representative biological replicate. The data for additional replicates are presented in Supplemental Figure S4. All differences in means relative to 0 hpi were assessed by a Dunnett’s test. Significance levels: * *p* < 0.005; *** *p* < 0.0005. (**B**) FHV-specific negative strand was detected at 72 hpi, which confirms that FHV productively infects primary honey bee hemocytes (Lane 1). Additional control reactions were performed using pooled RNA from time points 24–96 hpi (Lanes labeled 2–9). Specifically, RNA-isolated FHV containing cell lysate was reverse-transcribed with the primer listed below, treated with Exonuclease I to remove excess primer, and amplified using the PCR primers listed for each lane. (L) Molecular weight ladder. (1) 72 hpi RNA template—RT with FHV-TAGS-F, PCR with TAGS and FHV-Rev. (2) Negative control: No RT in the presence of (FHV-TAGS-F), PCR with TAGS and FHV-Rev. (3) Negative control: RT in the presence of (FHV-TAGS-F), PCR with only the FHV-Rev primer. (4) Negative control: RT with random hexamer primer, PCR with TAGS and FHV-Rev. (5) Negative control: No RT enzyme in the presence of random hexamers, PCR with FHV-F and FHV-Rev. (6) Positive control: RT with random hexamers, PCR with FHV-F and FHV-Rev. (7) Negative control: RT with random hexamers, PCR with only FHV-Rev primer. (8) Self-priming: RT without a primer, PCR with FHV-F and FHV-Rev. (9) Negative control: No template, no RT, PCR with TAGS and FHV-Rev. (10) Negative control: No template, no RT, PCR with FHV-F and FHV-Rev. Additional details provided in Supplemental Table S25.

To investigate the ability of honey bee immune cells to support the replication of a model virus, approximately 1 × 10^5^ larval-derived hemocytes maintained in culture were incubated with PBS or FHV (1 × 10^6^ FHV RNA copies). Cells were harvested at distinct time points post-infection (i.e., from 0 hpi to 96 hpi), and virus abundance was determined by qPCR. Data from two independent experiments demonstrated that FHV abundance increased from 0 hpi to 72 hpi and decreased from 72 hpi to 96 hpi (Supplemental Figure S4). In replicate one, FHV levels in hemocytes increased 3.7× by 24 hpi (*p* = 0.026) and 10.7× by 48 hpi, relative to 0 hpi (*p* < 0.001) (Figure 4A). FHV abundance continued to increase from 48 hpi to 72 hpi, which had 37× (*p* < 0.001) the initial FHV level (i.e., compared to 0 hpi) (Figure 4A). By 96 hpi, FHV abundance was less than levels quantified at 72 hpi (*p* = 0.034) but remained significantly higher relative to 0 hpi (*p* < 0.001) (Figure 4A). One possible explanation for reduced FHV abundance at 96 hpi is that, by that time most hemocytes were FHV-infected, there may have been a lack of naïve host cells to support further virus replication. In replicate two of this experiment, FHV abundance was constant at 24 hpi but was 16× higher at 48 hpi (*p* < 0.01) relative to 0 hpi. At 72 hpi, FHV levels peaked, with 39.8× higher FHV levels compared to 0 hpi (*p* < 0.001). FHV abundance was reduced at 96 hpi relative to 72 hpi but was still 12.6× higher that levels at 0 hpi (*p* < 0.001) (Supplemental Figure S4B). To further validate that FHV, which has a +ssRNA genome, productively infected cultured honey bee hemocytes, negative-strand-specific PCR was utilized to detect the replicative intermediate of FHV. Since FHV abundance peaked at 72 hpi, this time point was selected to assay for the presence of negative strand, and a negative-strand-specific FHV PCR product was detected (Figure 4B). Together, FHV abundance and negative-strand data demonstrate that FHV productively infects primary honey bee hemocytes maintained in culture.

The ability to infect hemocytes with FHV provides an additional tool for investigating virus–honey bee host interactions at the cellular level. This model virus may prove particularly useful in investigating RNAi in the context of viral suppressors, since FHV-B2 protein suppresses RNAi, as confirmed by studies that utilized deletion mutants (i.e., FHV-ΔB2) [128,191]. To date there have been relatively few studies on putative bee virus-encoded VSRs, and the results from IAPV-infected honey bees and bumble bees have mixed results regarding the presence of a VSR; therefore, additional investigation is required [150,190]. The proteins produced by other model insect viruses, including cricket paralysis virus and Drosophila C virus, which encode bona fide VSRs, have a conserved amino acid motif (DvExNPGP) downstream of their VSR proteins [192,193]. Sequence analysis indicates that this motif is present in IAPV, KBV, and ABPV, suggesting that these viruses may encode VSRs, although additional studies are needed to identify and validate putative VSRs. Overall, the use of both model viruses and naturally infecting honey bee viruses, including those that do and do not encode RNAi suppressors, will advance our knowledge of the interactions between viruses and the host antiviral RNAi machinery.

### 3.5. Flock House Virus Replicates in Primary Honey Bee Pupal Cells

**Figure 5:**
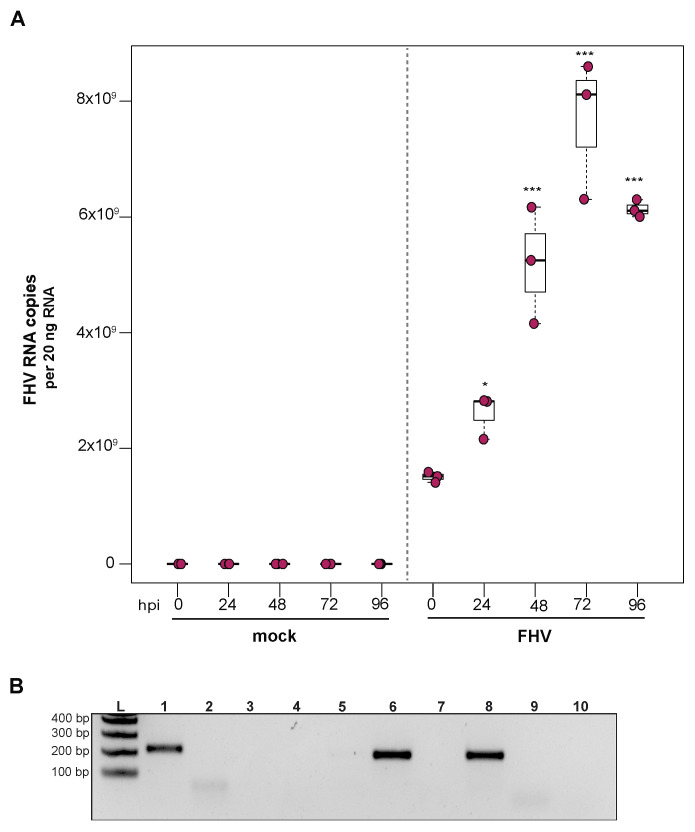
Flock House virus (FHV) replicates in primary honey bee pupal cells. Pupal cells were either mock- or FHV-infected (2 × 10^8^ FHV RNA copies), and FHV abundance was quantified over a course of time (i.e., 0 hpi, 24 hpi, 48 hpi, 72 hpi, and 96 hpi). (**A**) FHV abundance increased at 24 hpi (1.7×, *p* = 0.024) and 48 hpi (3.4× *p* < 0.001), with a peak in FHV infection at 72 hpi (5×, *p* < 0.001), relative to 0 hpi. FHV abundance did not increase from 72 hpi to 96 hpi but remained 4× higher than at 0 hpi (*p* < 0.001). Raw data are included in Supplemental Table S6. Figure 5 includes results from one representative biological replicate. The data for additional replicates are presented in Supplemental Figure S5. All differences in means relative to 0 hpi were assessed by a Dunnett’s test. Significance levels: * *p* < 0.05; *** *p* < 0.0005. (**B**) To validate that the increase in FHV abundance was indicative of virus replication, the presence of the negative strand (replicative intermediate) was assessed by negative-strand-specific RT followed by PCR. The negative strand was detected at 72 hpi (Lane 1)**.** Additional control reactions were performed using pooled RNA from time points 24–96 hpi (Lanes labeled 2–9). Specifically, RNA-isolated FHV containing cell lysate was reverse-transcribed with the primer listed below, treated with Exonuclease I to remove excess primer, and amplified using the PCR primers listed for each lane. (L) Molecular weight ladder. (1) 72 hpi RNA template—RT with FHV-TAGS-F, PCR with TAGS and FHV-Rev. (2) Negative control: No RT in the presence of (FHV-TAGS-F), PCR with TAGS and FHV-Rev. (3) Negative control: RT in the presence of (FHV-TAGS-F), PCR with only the FHV-Rev primer. (4) Negative control: RT with random hexamer primer, PCR with TAGS and FHV-Rev. (5) Negative control: No RT enzyme in the presence of random hexamers, PCR with FHV-F and FHV-Rev. (6) Positive control: RT with random hexamers, PCR with FHV-F and FHV-Rev. (7) Negative control: RT with random hexamers, PCR with only FHV-Rev primer. (8) Self-priming: RT without a primer, PCR with FHV-F and FHV-Rev. (9) Negative control: No template, no RT, PCR with TAGS and FHV-Rev. (10) Negative control: No template, no RT, PCR with FHV-F and FHV-Rev. Additional details provided in Supplemental Table S25.

To examine the ability of cells derived from purple-eyed honey bee pupae to support infection and replication of FHV, approximately 1 × 10^6^ primary cells in culture were incubated with FHV (2 × 10^8^ FHV RNA copies), and virus abundance was assessed at distinct time points post-infection. FHV abundance increased from 0 hpi to 72 hpi and was reduced or remained constant at 96 hpi in two experimental replicates. In replicate one, FHV levels increased by 1.7× at 24 hpi (*p* = 0.024) and by 3.4× at 48 hpi, relative to initial levels at 0 hpi (*p* < 0.001). The peak of infection was reached at 72 hpi, with 5× greater FHV levels relative to 0 hpi (*p* < 0.001). FHV abundance in pupal cells did not increase from 72 hpi to 96 hpi but remained 4× higher than at 0 hpi (*p* < 0.001) (Figure 5A). Similarly, in the second biological replicate, FHV abundance was 2.6× higher at 24 hpi (*p* = 0.013) and 7.5× higher at 48 hpi (*p* < 0.001), relative to levels at 0 hpi. FHV abundance increased 20× (*p* < 0.001) at 72 hpi relative to 0 hpi, which was the peak of virus infection. Similar to FHV infection in hemocytes, FHV levels in pupal cells was lower at 96 hpi relative to 72 hpi (*p* < 0.01) but was still 15.8× higher at 96 hpi compared to 0 hpi (*p* < 0.001) (Supplemental Figure S5B). To validate that the increase in FHV abundance was indicative of virus replication, the presence of FHV negative strand was assayed using strand-specific PCR. Active replication of FHV was confirmed by detection of FHV negative-strand PCR product at 72 hpi (Figure 5B). Together, the increase in FHV abundance quantified over the course of infections and the detection of the replicative intermediate form (i.e., negative strand) of FHV demonstrate that primary honey bee cells derived from purple-eyed pupae support the replication of FHV.

The ability to infect mixed cell types derived from honey bee pupae with a model virus helps address some challenges in studying honey bee host–virus interactions using naturally infecting honey bee viruses. FHV can be easily propagated and purified in the lab, which helps circumvent the problem of mixed virus inoculum produced from infected bees, since many honey bee viruses co-purify due to their similar size and the high prevalence of mixed infections in colonies [10,40,52,63,64,97]. Since FHV does not naturally infect honey bees, the experimental results may not be confounded by pre-existing infections. However, a limitation of using FHV as a model virus to investigate honey bee antiviral defense mechanisms is that it does not naturally infect honey bees and therefore may not induce the same broad-spectrum antiviral or virus-specific immune responses that occur during infection with a co-evolved virus. One benefit to utilizing FHV in studies aimed at elucidating honey bee antiviral defense is that FHV–host interactions have been studied in a broad range of insect hosts, including mosquitoes, fruit flies, honey bees, tsetse flies, and reduviid bugs, thus facilitating comparative immune studies [105,106,110,111].

### 3.6. Flock House Virus Infection Transferred from Infected to Naïve Honey Bee Pupal Cells

**Figure 6:**
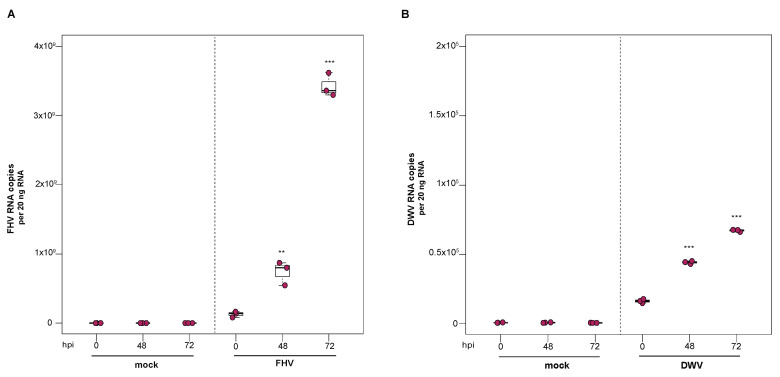
DWV and FHV infection transferred from infected to naïve honey bee pupal cells. To confirm that infectious DWV and FHV virions were produced in infected primary honey bee pupal cells, mock- or virus-infected honey bee pupal cell cultures (10 µL) at 72 hpi were transferred to naïve pupal cells. Virus abundance was assessed over a time course (i.e., 0 hpi, 48 hpi, and 72 hpi) using qPCR. Raw data are included in Supplemental Tables S7 and S8. (**A**) FHV abundance increased 15.8× at 48 hpi (*p* = 0.003), with a subsequent 28× increase at 72 hpi relative to 0 hpi (*p* < 0.001). (**B**) DWV abundance was 2.7× (*p* < 0.001) higher at 48 hpi and 4.1× higher at 72 hpi, relative to 0 hpi (*p* < 0.001). Figure 6 includes results from one representative biological replicate. The data for additional replicates are presented in Supplemental Figure S6. All differences in means relative to 0 hpi were assessed by a Dunnett’s test. Significance levels: ** *p* < 0.005; *** *p* < 0.0005.

To determine if FHV infection can be transferred from FHV-infected honey bee pupal cells to naïve cells, virus-containing cell culture obtained during peak infection (i.e., 72 hpi) was transferred to naïve pupal cells. Virus abundance in the secondary host cells was monitored over time. In replicate one, a small volume of FHV-infected pupal cell culture (i.e., 10 µL with 2.6 × 10^8^ FHV RNA copies) was transferred to freshly seeded pupal cells (Figure 6A). FHV abundance at 48 hpi was 15.8× greater (*p* = 0.003) and at 72 hpi 28× greater than levels at 0 hpi (*p* < 0.001). In the second replicate, FHV-infected pupal cells (i.e., 10 µL infected cell culture with 2.9 × 10^8^ FHV RNA copies) from the first round of infection were transferred to naïve pupal cells, and FHV abundance remained constant from 0 hpi to 48 hpi but was 10.4× higher at 72 hpi (*p* < 0.001) (Supplemental Figure S6B). As expected, FHV was not detected in control experiments, in which a small volume of mock-infected honey bee pupal cell culture was transferred to naïve cells (Figure 6A and Figure S6B). Together these results confirm that FHV virions produced by primary honey bee pupal cell cultures can be utilized to established new/secondary infections (Figure 6A).

### 3.7. Deformed Wing Virus Infection Transferred from Infected to Naïve Honey Bee Pupal Cells

To determine if the DWV-infected cultured primary honey bee pupal cells produced infectious virions, small volumes of DWV-infected cell cultures were transferred to naïve cultured pupal cells. In replicate one, naïve honey bee pupal cells were treated with cells and cell culture supernatant (10 µL) from either mock-infected or DWV-infected cultures, which contained an equivalent of 1.04 × 10^5^ DWV RNA copies. By 48 hpi, DWV abundance increased 2.7× relative to 0 hpi (*p* < 0.001). DWV levels continued to increase over time, with 4.1× increase at 72 hpi (*p* < 0.001) (Figure 6B). In replicate two, naïve pupal cells were treated with 10 µL DWV-infected pupal cells (i.e., 9 × 10^4^ DWV RNA copies). DWV abundance increased by 2.3× at 48 hpi (*p* < 0.01) and was 10.4× greater at 72 hpi, relative to 0 hpi (*p* < 0.001) (Supplemental Figure S6D). As expected, DWV was not detected in mock-infected cells, in which a small volume of PBS-treated honey bee pupal cell culture was transferred to naïve cells. The increase in DWV abundance over time and detection of negative strand suggest that the DWV infection in honey bee pupal cells results in the production of infectious DWV virions that have the ability to infect naïve host cells.

### 3.8. Immune Gene Expression in Virus-Infected Primary Honey Bee Cells

**Figure 7:**
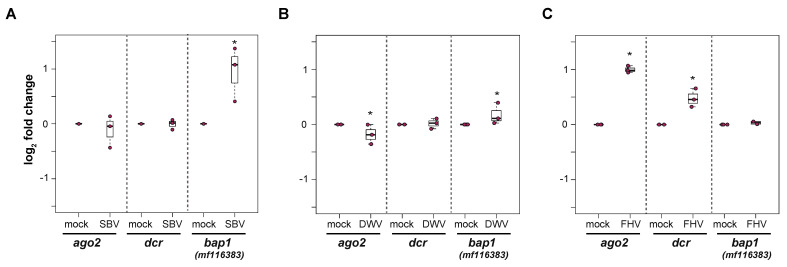
Virus infections of primary honey bee pupal cells differentially impact the expression of select immune genes. The relative expression of three honey bee immune genes, *argonaute-2 (ago2)*, *dicer-like (dcr-like)*, and *bee antiviral protein-1 (bap1)/mf116383*, were assessed by qPCR at 72 hpi. The ΔΔCt method with normalization to *rpl8* was utilized to assess target gene expression in each sample, and then the log_2_ fold change values were calculated relative to mock-infected samples. Raw data are included in Supplemental Tables S9–S11. (**A**) SBV infection of pupal cells did not impact *ago2* or *dcr-like* expression, whereas the expression of *bap1* was 0.95 log_2_-fold greater at 72 hpi (*p* = 0.032). (**B**) DWV-infected pupal cells exhibited slightly lower *ago2* expression (*p* = 0.032) relative to mock-infected cells at 72 hpi, whereas there was no impact of virus infection on *dcr-like* expression. The expression of *bap1* was 0.18 log_2_-fold higher in DWV-infected pupal cells compared to mock-infected cells at 72 hpi (*p* = 0.032). (**C**) In pupal cells infected with FHV, *ago2* and *dcr-like* expression was higher (i.e., 1 and 0.48 log_2_-fold change, respectively, *p* = 0.032) in FHV-infected cells relative to mock-infected cells at 72 hpi. The expression of *bap1* was not impacted by FHV infection. The differences in log_2_ immune gene relative expression in infected cells relative to mock-infected (PBS-treated) cells at 72 hpi were assessed using a one-sided Wilcoxon Rank Sums test. Significance level: * *p* < 0.05.

Honey bee primary cells will be an important tool to investigate honey bee antiviral defense mechanisms. We hypothesized that differential transcription of immune genes in primary cultures will reflect the transcriptional response in individuals (i.e., pupae or adults) [86,100,101,107,108,145,146,190]. Therefore, to begin to characterize cellular-level honey bee antiviral immune responses, honey bee pupal cells were infected with SBV, DWV, or FHV, and the expression of select antiviral genes, including *dicer-like (dcr-like)*, *argonaute-2 (ago2)*, and *bee antiviral protein-1* (*bap1*), which was formerly designated as GenBank MF116383, were analyzed by qPCR in either two or three experimental replicates (Figure 7 and Supplemental Figures S7–S10).

Overall, the expression of *bap1* (GenBank: MF116383) was greater in SBV-infected cells than mock-infected pupal cells at 72 hpi. Specifically, in first biological replicate, *bap1* expression was 0.67 log_2_-fold greater in SBV-infected pupal cells relative to the expression level in mock-infected cells (Wilcoxon Rank Sums, *p* = 0.032) (Supplemental Figure S7C). Likewise, *bap1* expression was greater in the second biological replication (0.95 log_2_-fold, *p* = 0.032, Figure 7A) but not the third. Since the *bap1* expression was greater two of the three biological replicates of SBV-infected pupal cells, its expression in the third biological replicate was also assessed at 96 hpi, at which time its expression was 0.73 log_2_-fold higher in SBV-infected cells relative to mock-infected cells (*p* = 0.032) (Supplemental Figure S7C). Expression of neither *ago2* nor *dcr-like* were appreciably impacted by SBV infection. Specifically, *ago2* was not differentially expressed in the first two replicates but had −0.76 log_2_-fold lower expression in the third replicate (*p* = 0.032) (Figure 7A and Figure S7A). Similarly, *dcr-like* was not differentially expressed in the second replicate but exhibited −0.67 log_2_-fold lower expression in the third replicate (*p* = 0.032) (Supplemental Figure S7). In DWV-infected pupal cells, *bap1* expression was unchanged in one replicate (Supplemental Figure S8C) and slightly greater in the second experimental replicate relative to mock-infected cells at 72 hpi (0.18 log_2_-fold change, *p* = 0.032, Figure 7B). In DWV-infected pupal cells, *ago2* expression was slightly lower compared to mock-infected cells in two biological replicates (−0.28 and −0.18 log_2_-fold change, *p* = 0.032, Figure 7B and Figure S8A). In contrast, *dcr-like* expression was slightly lower in DWV-infected pupal cells in one replicate (−0.26 log_2_-fold, *p* = 0.032, Figure S8B), while its expression was similar to mock-infected cells in another replicate (Supplemental Figure S7B). The expression of *bap1* was not appreciably impacted by FHV infection of pupal cells (Figure 7C and Figure S9C). Specifically, *bap1* expression was slightly lower in one replicate (−0.4 log_2_-fold, *p* = 0.032) and unchanged in the second replicate, compared to mock-infected cells (Figure 7C and Figure S9C). Interestingly, *ago2* expression was consistently higher in FHV-infected cells relative to mock-infected cells in two replicates (i.e., 1 log_2_-fold in rep1 and 1 log_2_-fold in rep2, *p* = 0.032) (Figure 7C and Figure S9A). Similarly, *dcr-like* expression was greater in FHV-infected cells relative to mock-infected cells in both replicates (i.e., 0.66 log_2_-fold in rep1 and 0.48 log_2_-fold in rep2, *p* = 0.032) (Figure 7C and Figure S9B). The trends in immune gene expression in FHV-infected hemocytes were similar to FHV-infected pupal cells (Supplemental Figure S10). Specifically, *bap1* expression was similar in FHV-infected and mock-infected cells at 72 hpi, whereas FHV-infected hemocytes exhibited greater *ago2* (i.e., 1.1 log_2_-fold in rep1 and 0.54 log_2_-fold in rep2, *p* = 0.032) and *dcr-like* expression (i.e., 0.45 log_2_-fold in rep1 and 0.63 log_2_-fold in rep2, *p* = 0.032) than mock-infected hemocytes in both biological replicates (Supplemental Figure S10).

Together these experiments indicate that studies in cultured honey bee primary cells recapitulate data obtained from transcriptome level studies in individual honey bees, including those carried out in pupae and adult bees [100], though additional studies are needed to better assess honey bee antiviral immune responses at the cellular level. Further development, including the advancements described herein, and utilization of honey bee primary cells is integral to elucidating the defense pathways important for specific virus infections and, in turn, identifying viral counter defense strategies.

## 4. Conclusions

In summary, these data indicate that primary honey bee cell cultures of larval hemocytes and explanted pupal tissue serve as a useful tool for studying honey bee–host interactions. In addition to demonstrating that a panel of viruses (SBV, DWV, and FHV) replicates in hemocytes and/or pupal cells, pupal cell cultures showed virus-specific transcriptional regulation of three immune genes (*dcr-like*, *ago2,* and *bap1*). These cultures can be established using rather simple techniques that are amenable to higher sample size studies. Future work to optimize transfection in these different cell culture types will be paramount for performing more advanced mechanistic studies. This includes utilizing RNAi-mediated gene knockdown, which facilitates the study of honey bee host–virus interactions by directly demonstrating the importance of specific genes in limiting particular viruses. For example, this study indicates that hemocytes are a natural site of infection for SBV and that SBV results in higher *bap1* expression in infected pupal cells. By reducing the expression of *bap1* in cell cultures that are infected with SBV, one could test the hypothesis that this gene is important for cells to combat SBV infection. Additionally, the use of cell culture will facilitate the study of basic virological questions, including virus cellular tropism, entry, and uncoating mechanisms. Additional efforts to establish virus-free cell lines from explanted honey bee tissues will prove useful for the production of pure virus stocks, especially in concert with the development of cDNA clones capable of producing infectious virus. The data presented in this study represent one of many steps toward the development of sophisticated cell culture techniques for the study of honey bee host–virus interactions.

## Data Availability

The majority of the data generated or analyzed during this study are included in this published article and its Supplementary Material files (available online). Additional data are available from the corresponding author upon request.

## Supplementary Materials

The following are available online at https://www.mdpi.com/article/10.3390/insects12070653/s1, Excel file with Supplementary Tables S1–S24, and Supplemental Figures S1–S12. Supplemental Table and Supplemental Figure titles are listed below: Supplemental Table S1. Primers used in this study. Supplemental Table S2. Raw data for SBV-infected hemocytes (Figure 1). Supplemental Table S3. Raw data for SBV-infected purple-eyed pupal cells (Figure 2 and Figure S2). Supplemental Table S4. Raw data for DWV-infected purple-eyed pupal cells (Figure 3 and Figure S3). Supplemental Table S5. Raw data for FHV-infected hemocytes (Figure 4 and Figure S4). Supplemental Table S6. Raw data for FHV-infected purple-eyed pupal cells (Figure 5 and Figure S5). Supplemental Table S7. Raw data for transfer of DWV infection from DWV-infected purple-eyed pupal cells to naïve purple-eyed pupal cell (Figure 6 and Figure S6). Supplemental Table S8. Raw data for transfer of FHV infection from FHV-infected purple-eyed pupal cells to naïve purple-eyed pupal cells (Figure 6 and Figure S6). Supplemental Table S9. Raw Ct values for immune gene expression in SBV-infected pupal cells (Figure 7 and Figure S7). Supplemental Table S10. Raw data for immune gene expression in DWV-infected honey bee pupal cells (Figure 7 and Figure S8). Supplemental Table S11. Raw data for immune gene expression in FHV-infected honey bee pupal cells (Figure 7 and Figure S9). Supplemental Table S12. Raw data for immune gene expression in FHV-infected hemocytes (Figure S10). Supplemental Table S13. SBV fold change relative to 0 hpi in hemocytes. Supplemental Table S14. SBV fold change relative to 0 hpi in pupal cells. Supplemental Table S15. DWV fold change relative to 0 hpi in purple-eyed pupal cells. Supplemental Table S16. FHV fold change relative to 0 hpi in hemocytes. Supplemental Table S17. FHV fold change relative to 0 hpi in purple-eyed pupal cells. Supplemental Table S18. DWV fold change relative to 0 hpi in naïve pupal cells treated with material from infected cells. Supplemental Table S19. FHV fold change relative to 0 hpi in naïve pupal cells treated with material from infected cells. Supplemental Table S20. Fold change and *p*-values for immune genes in SBV-infected pupae. Supplemental Table S21. Immune gene fold change and *p*-values for DWV-infected honey bee pupal cells. Supplemental Table S22. Immune gene fold change and *p*-values for FHV-infected honey bee pupal cells. Supplemental Table S23. Immune gene fold change and *p*-values for FHV-infected hemocytes. Supplemental Table S24. Virus testing in corresponding honey bee larval carcass and hemocyte samples indicate that hemocytes are natural sites of infection for black queen cell virus (BQCV). Supplemental Table S25. Negative strand-specific RT-PCR confirms viral replication in hemocytes and pupal cells. Supplemental Figure S1. Larval hemocyte cultures have relatively few contaminating adipocytes. Supplemental Figure S2. Sacbrood virus (SBV) replicates in primary honey bee pupal cell cultures. Supplemental Figure S3. Deformed wing virus (DWV) replicates in primary honey bee pupal cell cultures. Supplemental Figure S4. Flock House virus (FHV) replicates in primary honey bee hemocytes. Supplemental Figure S5. Flock House virus (FHV) replicates in primary honey bee pupal cells. Supplemental Figure S6. DWV and FHV infection transferred from infected to naïve honey bee pupal cells. Supplemental Figure S7. Infection of primary pupal cells with SBV results in higher *bap-1* expression. Supplemental Figure S8. Infection of primary honey bee pupal cells with DWV has modest impact on the expression of select immune genes. Supplemental Figure S9. Infection of primary honey bee pupal cells with FHV results in differential regulation of immune genes. Supplemental Figure S10. Infection of honey bee hemocytes with FHV results in differential regulation of immune genes. Supplemental Figure S11. Sacbrood virus (SBV) inoculum was free of several other common honey bee infecting viruses. Supplemental Figure S12. Deformed wing virus (DWV) inoculum was free of several other common honey bee infecting viruses.
