# Supplementary material for: Investigating Virus–Host Interactions in Cultured Primary Honey Bee Cells
Source: Insects. 2021 Jul 17;12(7):653. doi: 10.3390/insects12070653 (PMC8329929; doi:10.3390/insects12070653)
Supplement: Supplementary file 1 [file insects-12-00653-s001.zip › SFigs S1-S12 June29 2021.pdf]

Supplemental Figure S1. Larval hemocyte cultures have relative few contaminating adipocytes. (A) Hemocytes were live-imaged at 20x magnification in complete medium on a Nikon Ti-Eclipse (Nikon Instruments) inverted microscope equipped with a SpectraX LED (Lumencor) excitation module and fast-switching emission filter wheels (Prior Scientific). Scale bar is 100 µm. (B) Hemocytes were live imaged at 100x magnification. Scale bar is 10 µm. (C) Cells were fixed in 4% paraformaldehyde and then nuclei were stained using Hoechst 33258 stain at 30 mg/mL, and images were captured at 20x magnification. Fluorescent images were captured using paired excitation/emission filters and dichroic mirrors for DAPI. From left to right: combined image, bright field image, and DAPI stain image. Scale bar is 100 µm. (D) Fixed images were captured using either bright field or fluorescent imaging at 100x magnification. Scale bar is 10 µm. Together these images show that the isolation technique utilized in this study results in relatively pure cultures of larval hemocytes with relatively little contaminating adipocytes.

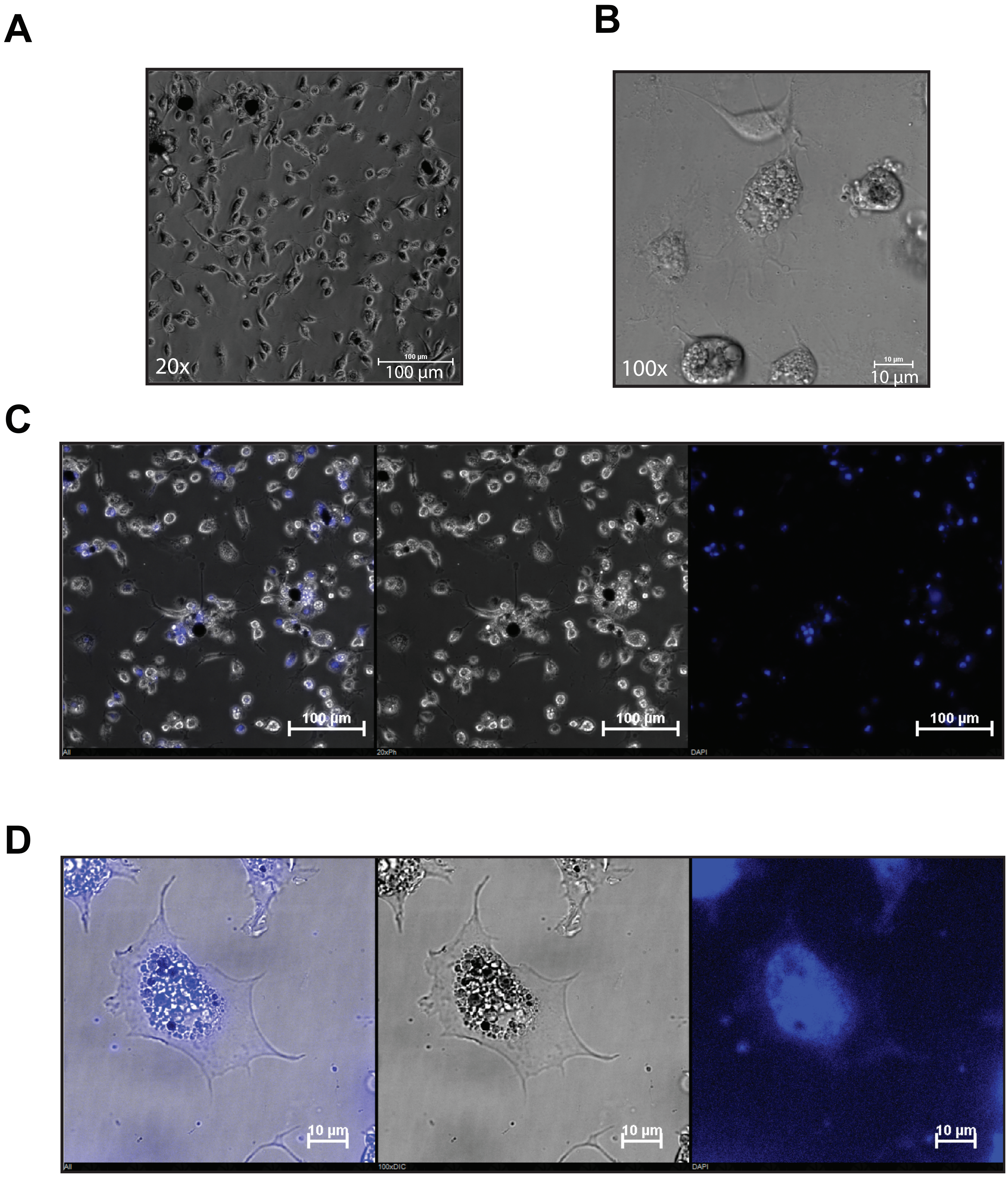

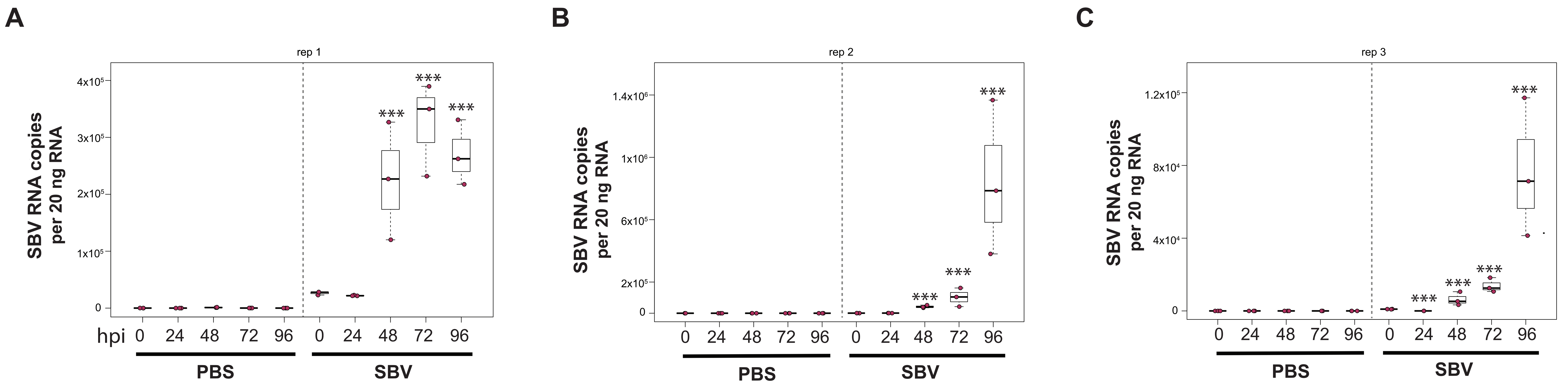

Supplemental Figure S2. Sacbrood virus (SBV) replicates in primary honey bee pupal cell cultures.
SBV abundance was assessed in mock- or SBV-infected pupal cells via qPCR over a time course (i.e., 0 hpi, 24 hpi, 48 hpi, 72 hpi, 96 hpi) in three biological replicates. (**A**) In replicate 1 pupal treated with a filtered larval lysate containing $2.5x10^6$ SBV RNA copies had 4.8x higher SBV by 48 hpi as compared to 0 hpi ($p < 0.0001$), which remained high at 72 hpi (12x, $p < 0.0001$) and 96 hpi (11.4x, $p < 0.0001$). (**B**) In replicate 2, SBV was significantly higher than 0 hpi by 48 hpi (59x, $p < 0.0001$) and remained high at 72 hpi (126x, $p < 0.0001$) and peaked at 96 hpi (1,047x, $p < 0.001$). This representative biological replicate is shown in Figure 2. (**C**) In replicate 3, SBV abundance at 24 hpi was lower (0.017x) than at 0 hpi ($p < 0.0001$). SBV abundance at 48 hpi was 2.2 x higher at 48 hpi relative to 0 hpi ($p = 0.0012$), as well as at 72 hpi (14x, $p = 0.00013$) and 96 hpi (72x, $p < 0.0001$). Overall (i.e., from the lowest at 24 hpi to the highest at 96 hpi) SBV abundance increased by 1,817x ($p < 0.0001$). Differences in means relative to 0 hpi were assessed by a Dunnett's test. Significance levels: * $p < 0.05$; ** $p < 0.005$; *** $p < 0.0005$.

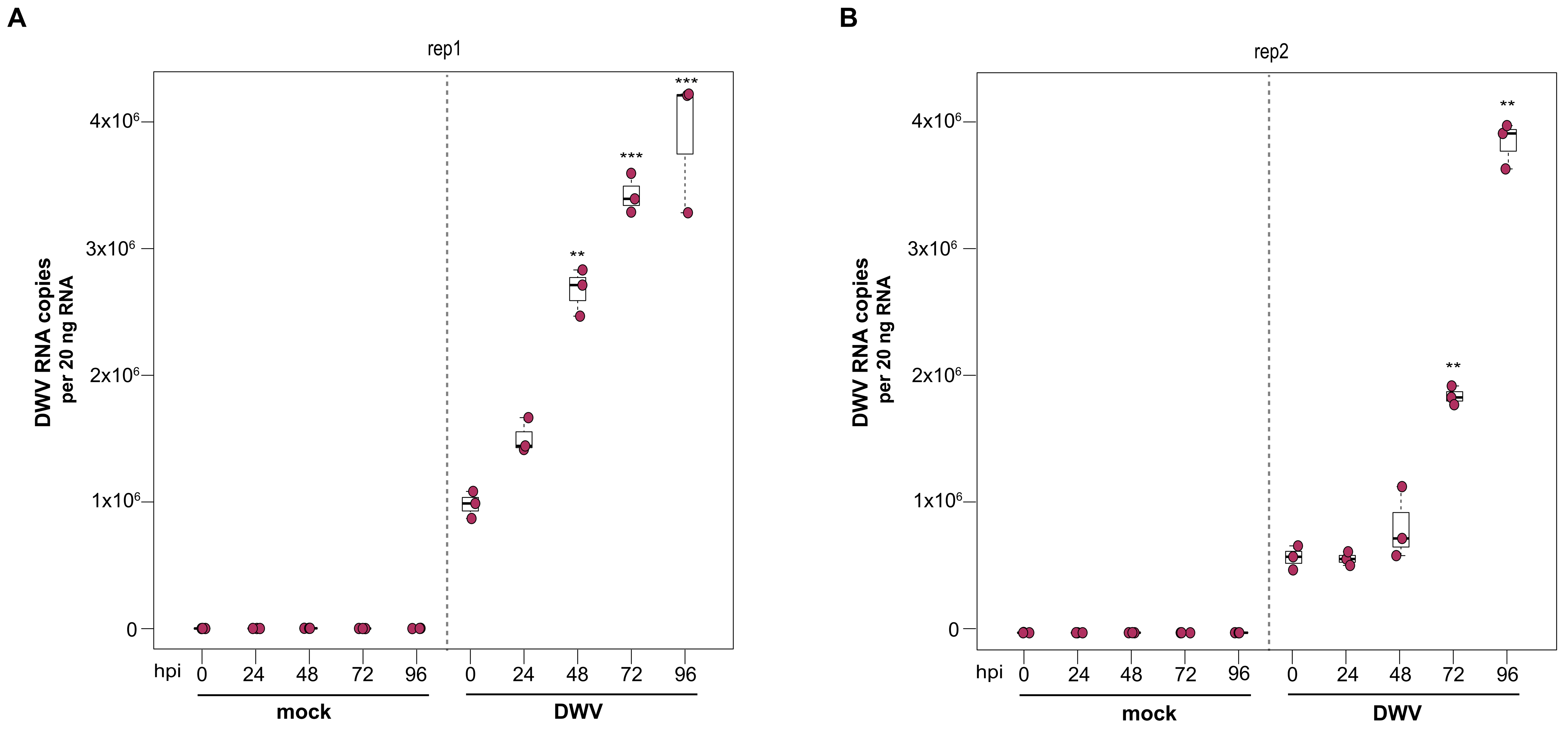

Supplemental Figure S3. Deformed wing virus (DWV) replicates in primary honey bee pupal cell cultures. DWV abundance was assessed in mock- or DWV-infected pupal cells via qPCR over a time course (i.e., 0 hpi, 24 hpi, 48 hpi, 72 hpi, 96 hpi) in two biological replicates. (**A**) In replicate 1, pupal cells infected with 4.3×10^6 DWV RNA copies had a 2.7x ($p < 0.001$) and 3.5x ($p < 0.001$) increase in mean DWV abundance relative to 0 hpi at 48 hpi and 72 hpi, respectively. At 96 hpi, mean DWV abundance was 4x higher relative to 0 hpi ($p < 0.001$). (**B**) In replicate 2, DWV levels remained similar to inoculum levels through 48 hpi, but were 3.2x higher at 72 hpi ($p = 0.001$), and 6.6x higher at 96 hpi, relative to 0 hpi ($p = 0.003$). One representative biological replicate (i.e., rep1) is shown in Figure 3. Differences in means relative to 0 hpi were assessed by a Dunnett's test. Significance levels: * $p < 0.05$; ** $p < 0.005$; *** $p < 0.0005$.

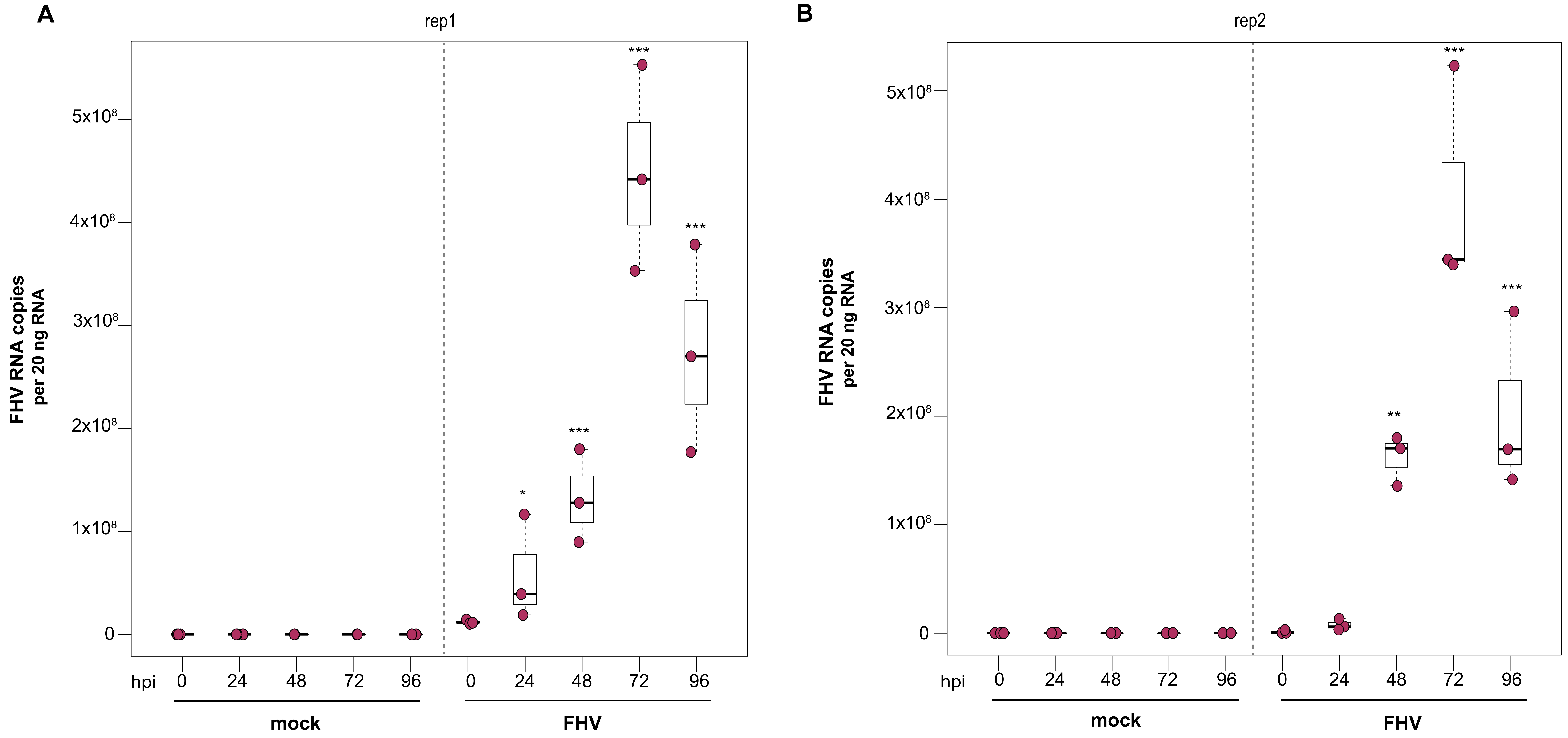

Supplemental Figure S4. Flock House virus (FHV) replicates in primary honey bee hemocytes.
FHV abundance in mock- or FHV- infected ($1x10^6$ FHV RNA copies) hemocytes was assessed over time (i.e., 0 hpi, 24 hpi, 48 hpi, 72 hpi, 96 hpi). (**A**) In replicate one, FHV abundance was higher at 24 hpi (3.7x, $p = 0.026$), 48 hpi (10.7x, $p < 0.001$) and 72 hpi (37x, $p < 0.001$) relative to 0 hpi. FHV abundance decreased from 72 hpi to 96 hpi ($p < 0.05$), but was significantly higher relative to 0 hpi ($p < 0.001$).
(**B**) In replicate two, FHV abundance at 24 hpi was similar to levels at 0 hpi. At 48 hpi FHV abundance was 16x higher than the 0 hpi ($p = 0.001$). FHV abundance peaked at 72 hpi, with 39.8x higher FHV levels compared to 0 hpi ($p < 0.001$). At 96 hpi, the abundance of FHV abundance was less than the level at 72 hpi, but was still 12.6x higher that levels at 0 hpi ($p < 0.001$). One representative biological replicate (i.e., rep1) is also shown in Figure 4. Differences in means relative to 0 hpi were assessed by a Dunnett's test.
Significance levels: * $p < 0.05$; ** $p < 0.005$; *** $p < 0.0005$.

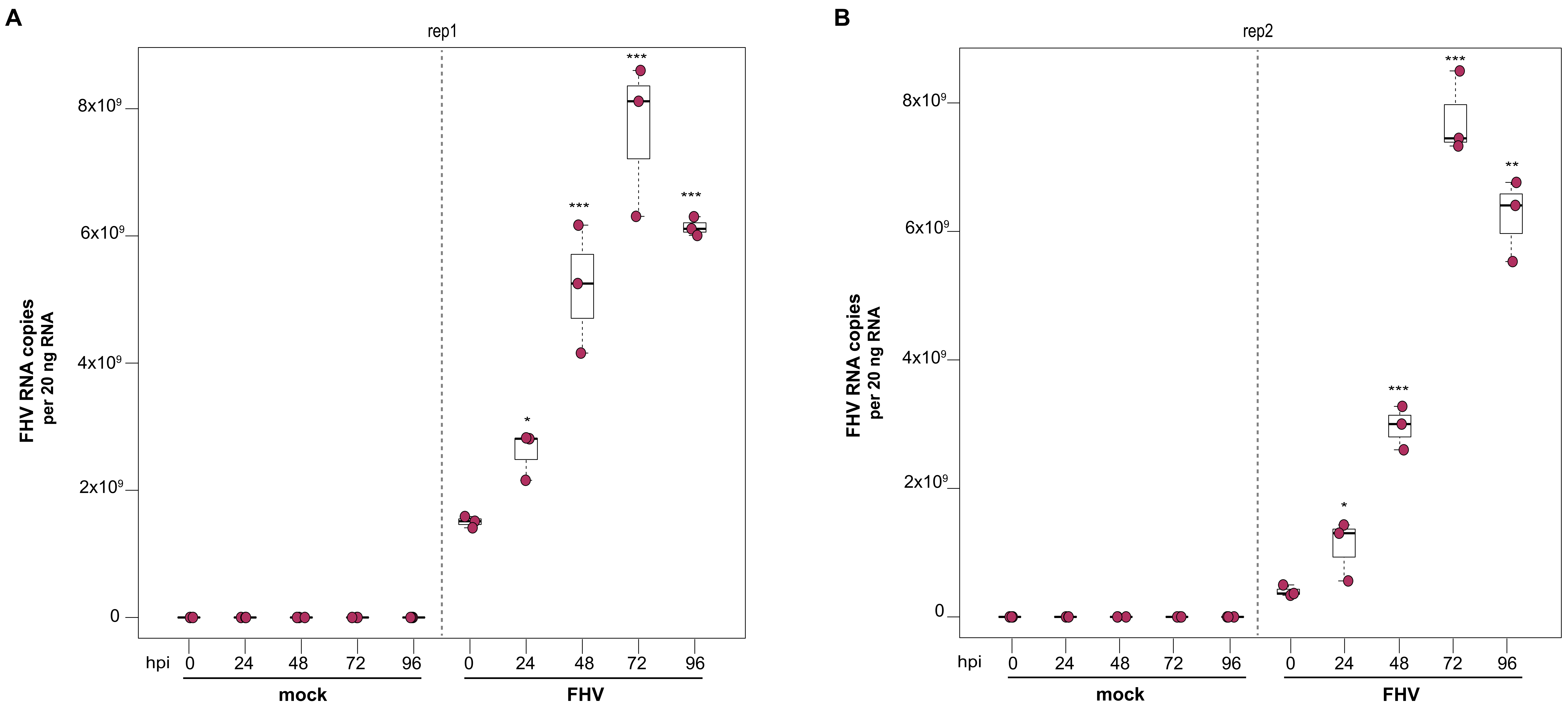

Supplemental Figure S5. Flock House virus (FHV) replicates in primary honey bee pupal cells.
Honey bee pupal cells were either mock- or FHV-infected ($2x10^8$ FHV RNA copies) and FHV abundance was quantified over a course of time (i.e., 0 hpi, 24 hpi, 48 hpi, 72 hpi, 96 hpi). (**A**) In replicate one, FHV abundance increased at 24 hpi (1.7x, $p = 0.024$) and 48 hpi (3.4x $p < 0.001$), with a peak in FHV infection at 72 hpi (5x, $p < 0.001$) relative to 0 hpi. FHV abundance did not increase from 72 hpi to 96 hpi, but remained 4x higher than the levels at 0 hpi ($p < 0.001$). (**B**) In replicate two, FHV abundance was higher at 24 hpi (2.6x, $p = 0.013$) and 48 hpi (7.5x, $p < 0.001$) relative to levels at 0 hpi. FHV abundance increased 20x at 72 hpi, which was the peak of virus infection ($p < 0.001$). FHV levels in pupal cells were lower at 96 hpi relative to 72 hpi ($p < 0.01$), but were still 15.8x higher at 96 hpi compared to 0 hpi ($p < 0.001$). One representative biological replicate (i.e., rep1) is shown in Figure 5. Differences in means relative to 0 hpi were assessed by a Dunnett's test.
Significance levels: * $p < 0.05$; ** $p < 0.005$; *** $p < 0.0005$.

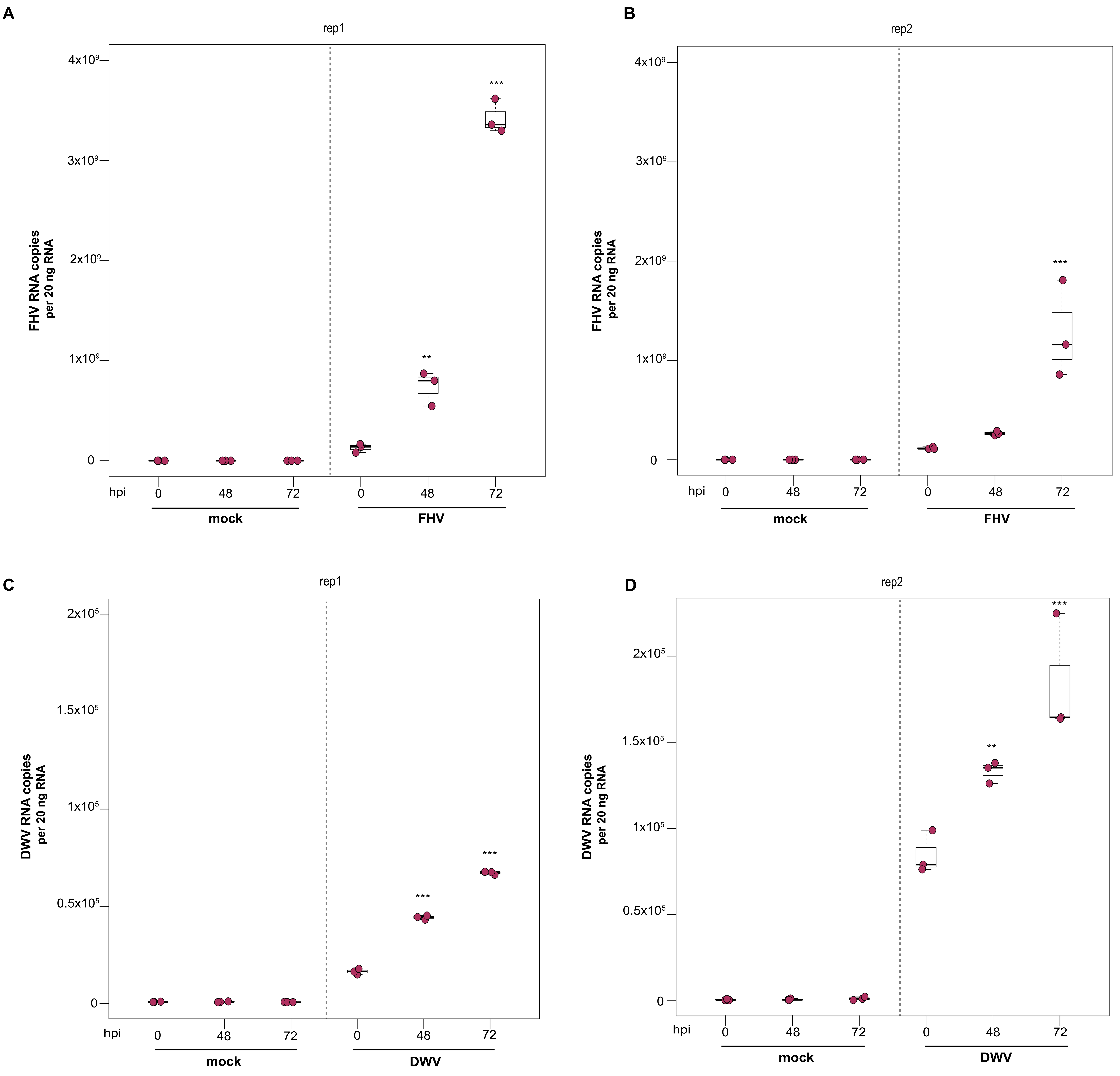

Supplemental Figure S6. DWV and FHV infection transferred from infected to naïve honey bee pupal cells.
Mock- or virus-infected honey bee pupal cell cultures at 72 hpi were utilized to transfer virus infection to naïve pupal cells. Virus abundance was assessed over a time course (i.e., 0 hpi, 48 hpi, 72 hpi) using qPCR. (**A**) In replicate one, naïve honey bee pupal cells were treated with either 10 µL of mock- or DWV-infected cells, which was an equivalent of $1.04x10^5$ DWV RNA copies. DWV abundance was 2.7x higher at 48 hpi compared to 0 hpi and 4.1x higher at 72 hpi relative to 0 hpi ($p < 0.001$). In replicate two, naïve pupal cells were treated with 10 µL of mock- or DWV-infected pupal cell culture, which contained 9x104 DWV RNA copies. DWV abundance increased by 2.3x at 48 hpi ($p = 0.002$) and was 10.4x greater at 72 hpi, relative to 0 hpi ($p < 0.001$). (**B**) In replicate one, 10 µL of mock- or FHV-infected pupal cell culture with 2.6 x 108 FHV RNA copies were utilized to infect naïve pupal cells. FHV abundance increased 15.8x at 48 hpi ($p = 0.003$) with a subsequent 28x increase at 72 hpi ($p < 0.001$) relative to 0 hpi. In replicate two, mock- or FHV-infected pupal cells (i.e., 10 µL infected cell culture with $2.9x10^8$ FHV RNA copies) from first round of infection were transferred to naïve pupal cells and FHV abundance remained constant from 0 hpi to 48 hpi but was 10.4x at 72 hpi ($p < 0.001$). One representative biological replicate (i.e., rep1) is shown in Figure 6. Differences in means relative to 0 hpi were assessed by a Dunnett's test. Significance levels: * $p < 0.05$; ** $p < 0.005$; *** $p < 0.0005$.

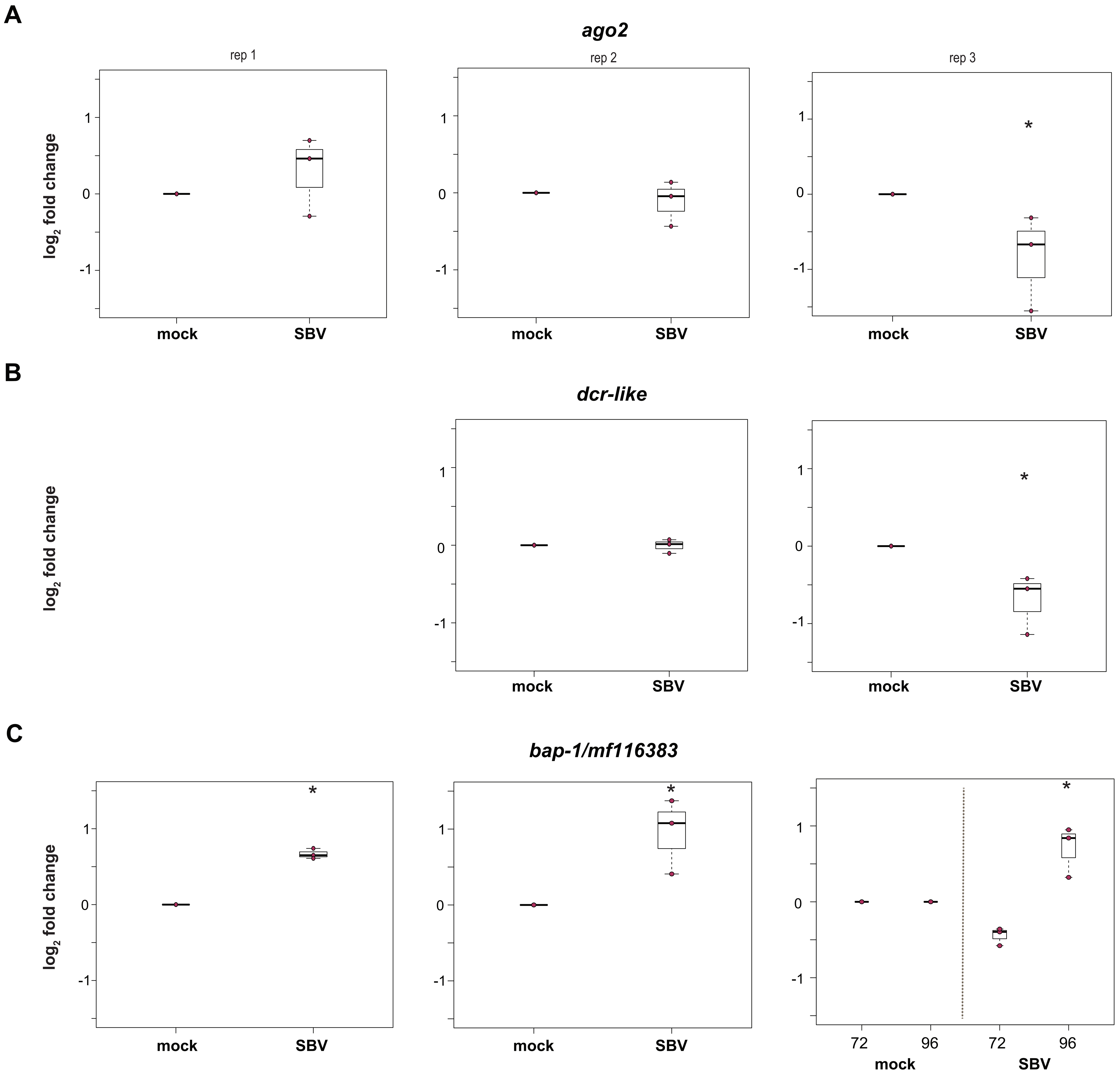

Supplemental Figure S7. Infection of primary pupal cells with SBV results in higher bap-1/mf116383 expression. The relative expression of three immune genes (i.e., *ago2, dcr-like*, and *bap1/mf116383*) in SBV-infected pupal cells was assessed by qPCR at 72 hpi relative to mock-infected cells. (**A**) In replicates 1 and 2, *ago2* expression levels were similar in SBV-infected and mock-infected cells, but in the third replicate *ago2* expression is significantly lower (-0.76 log_2-fold) in SBV-infected cells than mock at 72 hpi (p = 0.032). (**B**) While we were unable to collect *dcr-like* expression data in replicate 1, *dcr-like* expression was similar in SBV-infected and mock-infected cells in replicate 2. Similar to ago2, dcr-like expression in SBV-infected cells was less (-0.67 log_2-fold) than levels in mock-infected cells at 72 hpi (p = 0.032). (**C**) The relative expression of *bap1/mf116383* was higher in SBV-infected cells compared to mock-infected cells at 72 hpi (replicate 1 = 0.67 log2-fold, replicate 2 = 0.95 log_2-fold, p = 0.032). While levels of *bap1/mf116383* expression in SBV-infected and mock-infected cells were similar at 72 hpi in the third replicate, at 96 hpi, *bap1/mf116383* expression was higher (0.73 log_2-fold, p = 0.032) in SBV-infected cells than in mock-infected. Together, these data indicate SBV infection of honey bee pupal cells has little or no impact on *ago2* or *dcr-like* expression at 72 hpi, but does result in higher *bap1/mf116383* expression.

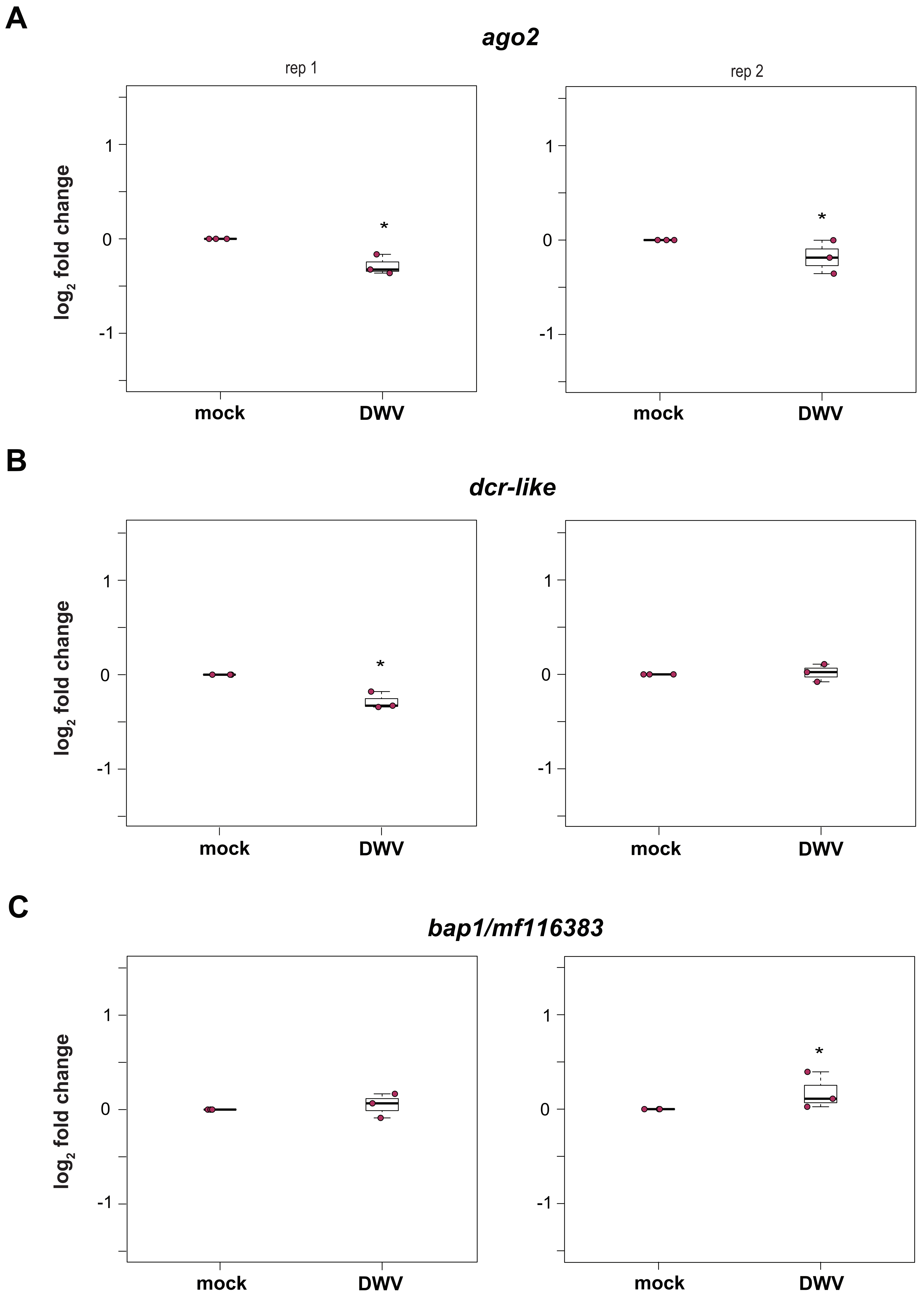

Supplemental Figure S8. Infection of primary honey bee pupal cells with DWV had modest impact on the expression of select immune genes. The expression of three honey bee immune genes (i.e., *ago2, dcr-like*, and *bap1/mf116383*) in DWV-infected pupal cells relative to mock-infected cells at 72 hpi was assessed by qPCR. (**A**) In both biological replicates (replicate one and two), *ago2* expression was lower in DWV-infected cells at 72 hpi relative to mock-infected cells ($p = 0.032$). (**B**) In replicate 1, the expression for dcr-like was lower in DWV-infected cells relative to mock-infected cells at 72 hpi ($p = 0.032$), but in replicate two, *dcr-like* expression was not impacted. (**C**) In replicate one, *bap1/mf116383* expression in DWV-infected and mock-infected cells at 72 hpi was similar, but in replicate two, the expression of *bap1/mf116383* was slightly higher in DWV-infected pupal cells compared to mock-infected cells ($p = 0.032$). One representative biological replicate (rep2) for each gene is shown in Figure 7. Differences in means relative to mock-infected cells were assessed by a one-sided Wilcoxon Rank Sums test.

Significance levels: * $p < 0.05$; ** $p < 0.005$; *** $p < 0.0005$.

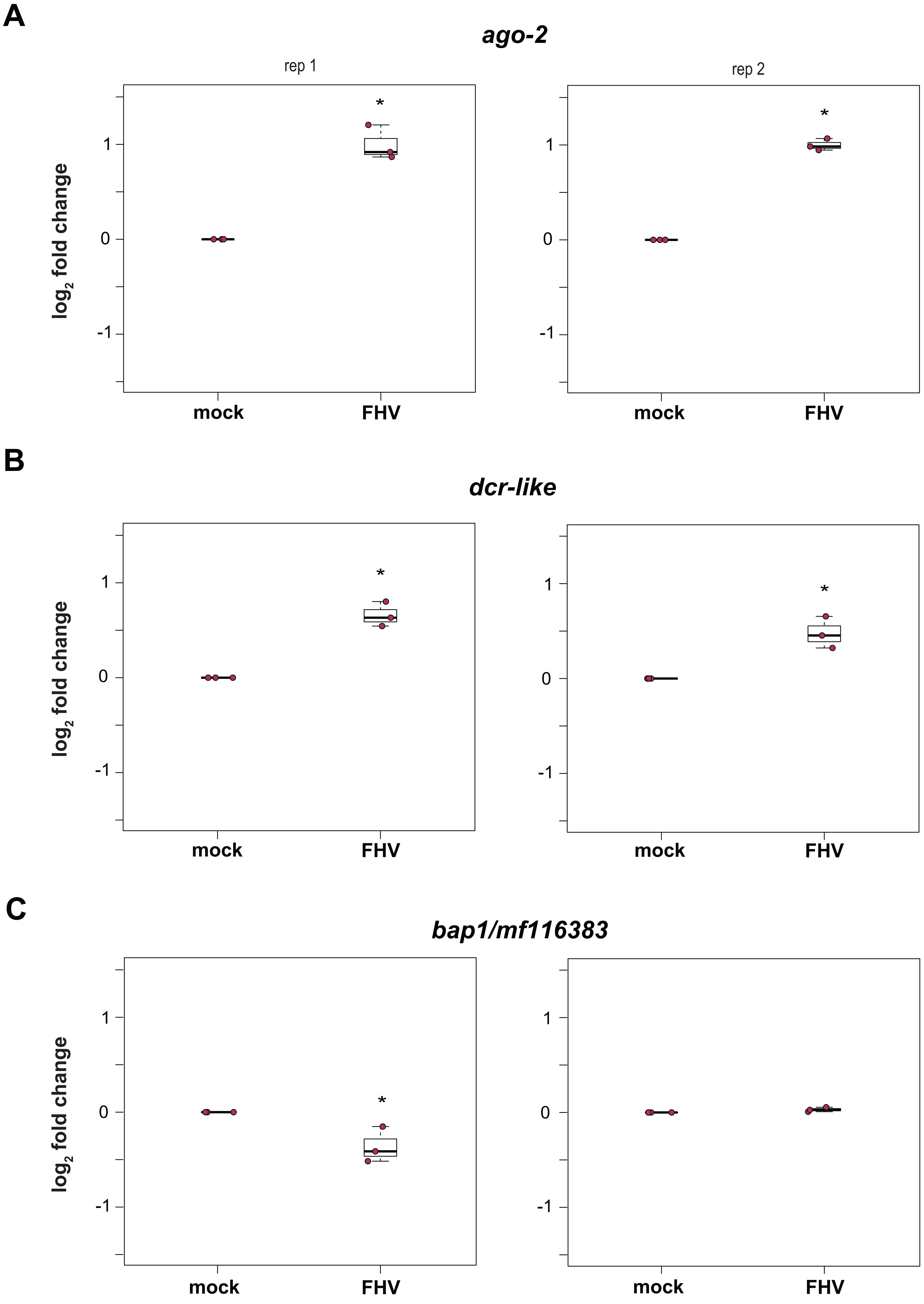

Supplemental Figure S9. Infection of primary honey bee pupal cells with FHV results in differential regulation of immune genes.
The expression of three immune genes (i.e., *ago2, dcr-like*, and *bap1/mf116383*) in FHV-infected pupal cells relative to mock-infected cells was assessed by qPCR at 72 hpi. (**A**) In both biological replicates (replicate one and two), *ago2* expression was higher in FHV-infected cells at 72 hpi relative to mock-infected cells ($p = 0.032$). (**B**) In replicate one and two, *dcr-like* expression was higher ($p = 0.032$) in FHV-infected cells relative to mock-infected cells at 72 hpi. (**C**) In replicate one, *bap1/mf116383* expression was slightly reduced in FHV-infected cells relative to mock-infected cells at 72 hpi ($p = 0.032$), but in replicate two, the levels of *bap1/mf116383* expression were similar in FHV-infected pupal cells compared to mock-infected cells at 72 hpi. One representative biological replicate (rep2) for each gene is shown in Figure 7. Differences in means relative to mock-infected cells were assessed by a one-sided Wilcoxon Rank Sums test. Significance levels: * $p < 0.05$; ** $p < 0.005$; *** $p < 0.0005$.

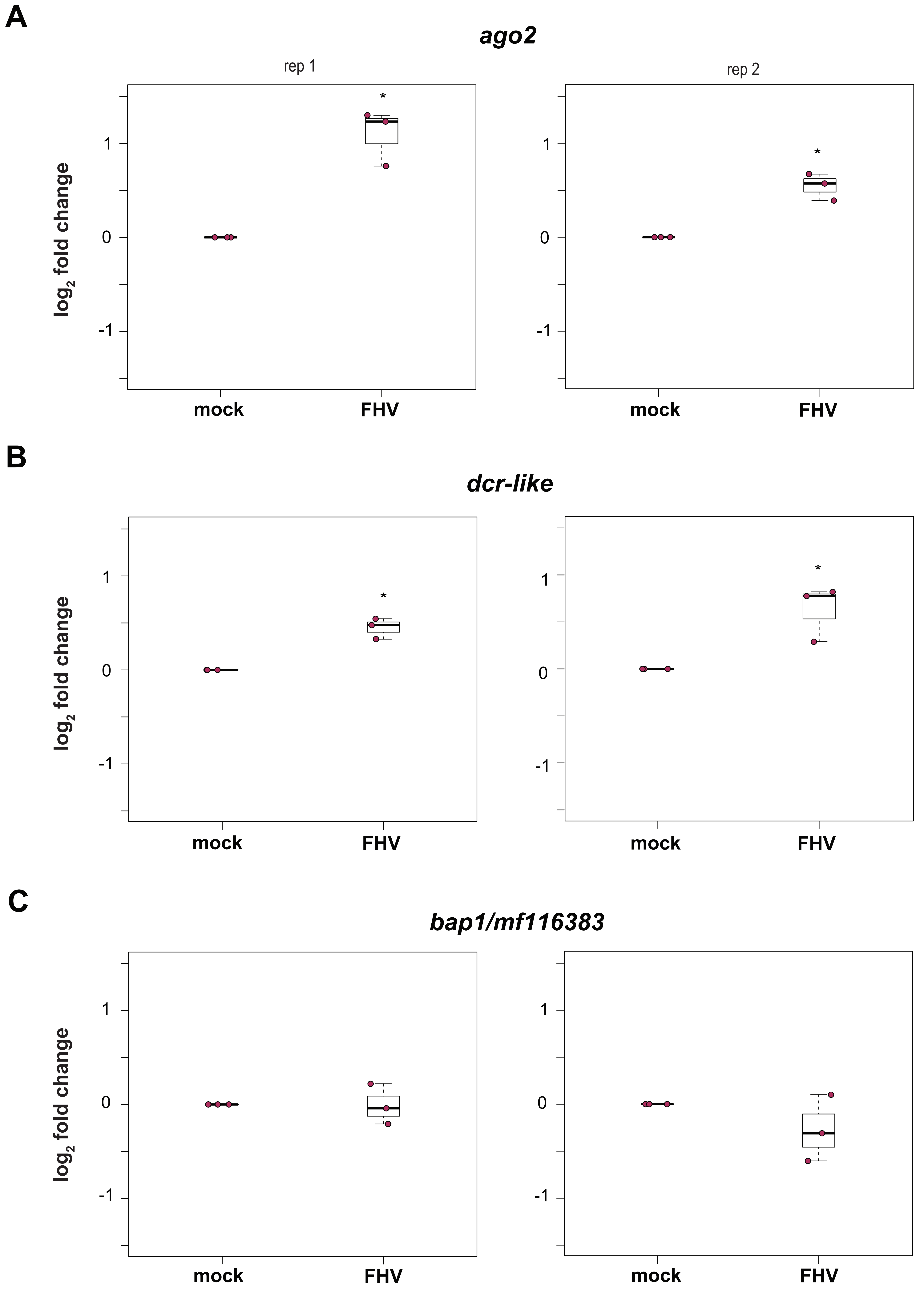

Supplemental Figure S10. Infection of honey bee hemocytes with FHV results in differential regulation of immune genes.
The expression of three immune genes (i.e., *ago2, dcr-like*, and *bap1/mf116383*) in FHV-infected hemocytes relative to mock-infected cells was assessed by qPCR at 72 hpi. (**A**) In both biological replicates (replicate one and two), *ago2* expression was higher in FHV-infected hemocytes at 72 hpi relative to mock-infected cells (i.e., 1.1 log_2-fold in rep1 and 0.54 log_2-fold in rep2, $p = 0.032$). (**B**) Likewise, *dcr-like* expression in FHV-infected hemocytes was higher compared to expression levels mock-infected cells at 72 hpi (i.e., 0.45 log_2-fold in rep1 and 0.63 log_2-fold in rep2, $p = 0.032$). (**C**) FHV infection did not have appreciable impact on *bap1/mf116383* expression at 72 hpi. Differences in means relative to mock-infected cells were assessed by a one-sided Wilcoxon Rank Sums test. Significance levels: * $p < 0.05$; ** $p < 0.005$; *** $p < 0.0005$.

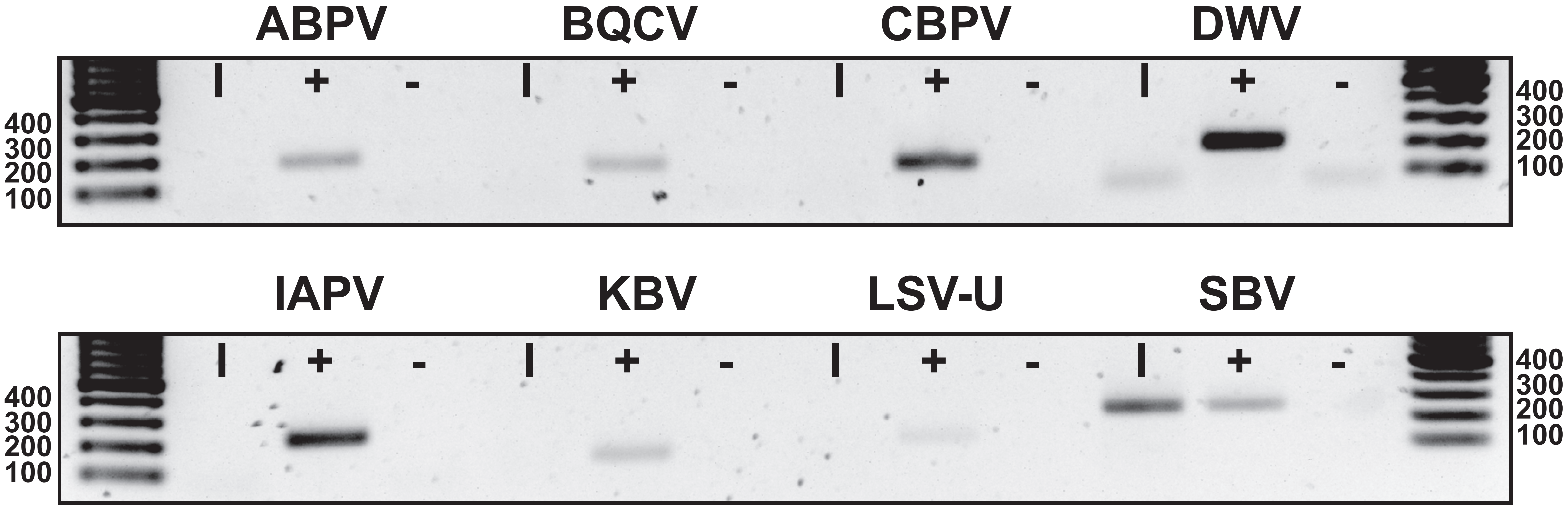

Supplemental Figure S11. Sacbrood virus (SBV) inoculum was free of several other common honey bee infecting viruses.
The SBV preparation that was used as inoculum in this study was tested for the presence of contaminating viruses using PCR. RNA was isolated from 100 µL SBV stock and reverse transcribed to synthesize cDNA. Pathogen specific PCR was carried out to test for the presence of acute bee paralysis virus (ABPV), black queen cell virus (BQCV), chronic bee paralysis virus (CBPV), deformed wing virus (DWV), Israeli acute paralysis virus (IAPV), Kashmir bee virus (KBV), Lake Sinai viruses (i.e., LSV1 and LSV2), and SBV, using primers listed in Supplemental Table S1. The inoculum was confirmed to have SBV and did not have detectable levels any of the other viruses tested.

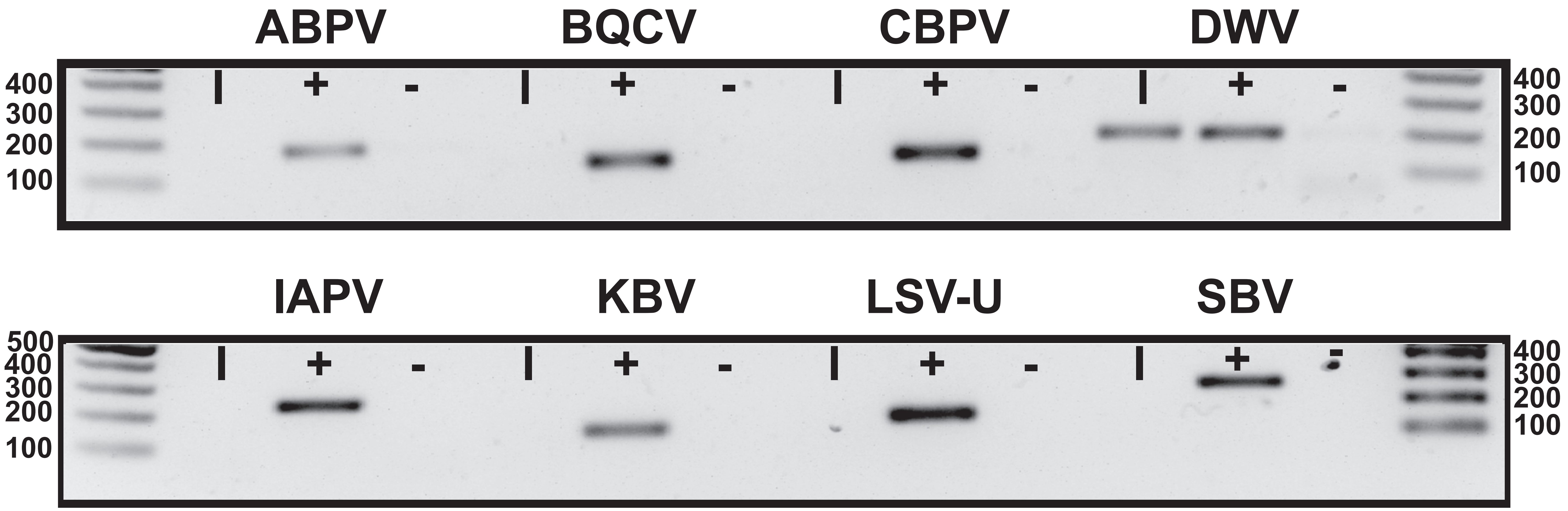

Supplemental Figure S12. Deformed wing virus (DWV) inoculum was free of several other common honey bee infecting viruses.
The DWV preparation that was used as inoculum in this study was tested for the presence of contaminating viruses using PCR. RNA was isolated from 100 µL DWV stock and reverse transcribed to synthesize cDNA. Pathogen specific PCR was carried out to test for the presence of acute bee paralysis virus (ABPV), black queen cell virus (BQCV), chronic bee paralysis virus (CBPV), Israeli acute paralysis virus (IAPV), Kashmir bee virus (KBV), Lake Sinai viruses (i.e., LSV1 and LSV2), sacbrood virus (SBV), and DWV, using primers listed in Supplemental Table S1. The inoculum was confirmed to have DWV and did not have detectable levels any of the other viruses tested.
